# Novel Isoquinoline Alkaloid Litcubanine A - A Potential Anti-Inflammatory Candidate

**DOI:** 10.3389/fimmu.2021.685556

**Published:** 2021-06-07

**Authors:** Huan Xia, Yitong Liu, Guiyang Xia, Yi Liu, Sheng Lin, Lijia Guo

**Affiliations:** ^1^ Key Laboratory of Chinese Internal Medicine of Ministry of Education and Beijing, Dongzhimen Hospital, Beijing University of Chinese Medicine, Beijing, China; ^2^ Laboratory of Tissue Regeneration and Immunology and Department of Periodontics, Beijing Key Laboratory of Tooth Regeneration and Function Reconstruction, School of Stomatology, Capital Medical University, Beijing, China; ^3^ Department of Orthodontics School of Stomatology, Capital Medical University, Beijing, China

**Keywords:** macrophages, anti-inflammatory activity, molecular mechanism, *Litsea cubeba*, isoquinoline *N*-oxide

## Abstract

Macrophages play a critical role in innate and adaptive immunity, and the regulation of macrophage function in inflammatory disease treatment has been widely studied. *Litsea cubeba* is an important Chinese medicinal plant used for the treatment of inflammatory diseases. However, the inflammatory bioactive ingredients in *L. cubeba* and underlying molecular mechanisms are poorly understood. Herein, we first obtained and elucidated a novel isoquinoline alkaloid, Litcubanine A (LA), from *L. cubeba*. An *in vitro* study indicated that LA could significantly inhibit LPS-induced activation of inflammatory macrophages *via* the NF-κB pathway, leading to the decrease of inflammatory factors including iNOS, TNF-α, and IL-1β. Moreover, LA showed an inhibiting effect on the expression of NO in macrophages by directly binding to iNOS protein. Molecular simulation docking also demonstrated that active LA created an interaction with GLU 371 residue of iNOS *via* attractive charge derived from the N→O group, revealing its highly selective inhibition toward iNOS. By using the IκK inhibitor and iNOS inhibitor, these two regulatory targets of LA on inflammatory macrophages were verified *in vitro*. Finally, by using a caudal fin resection model in zebrafish larvae, and the skin wound healing model in mice, we proved *in vivo* that LA down-regulated the secretion of local inflammatory factors by inhibiting macrophage recruitment and activation at the early stage of the injury. Collectively, our study demonstrated that the novel isoquinoline alkaloid LA suppresses LPS-induced activation of inflammatory macrophages by modulating the NF-κB pathway, suggesting that inflammatory macrophage activation pathway is an effective target for inflammation treatment, and LA is a new pharmacophore for the development of novel and effective anti-inflammatory agents to regulate local macrophages.

## Introduction

Macrophages have been identified as the key factor in the progression of tissue inflammation. Different phenotypes of macrophages exert diverse effects in inflammatory response and tissue remodeling (1–3). To date, at least two different phenotypes of macrophages have been reported: the classically activated macrophages (M1/inflammatory macrophages), and the alternatively activated macrophages (M2/anti-inflammatory macrophages). Inflammatory macrophages normally can be reduced by IFN-γ and lipopolysaccharide (LPS) through a classic nuclear factor kappa-B (NF-κB) signaling pathway. Inflammatory cytokines like LPS induce the phosphorylation of the inhibitor of kappaB (IκB) and IκB kinase (IκK), which promotes P65 nuclear translocation, leading to inflammatory macrophage activation. Inflammatory macrophages perform chemotaxis and become activated in the early stage of inflammation; they play a predominant role and enhance the inflammatory response by producing pro-inflammatory factors, like interleukin (IL)-1, TNF-α, IL-6, IL-23, reactive oxygen species (ROS), nitric oxide (NO), and inducible NO-synthase (iNOS) (4). An excess of local inflammatory macrophages may promote inflammation and contribute to tissue injury and destruction (1). In contrast, IL-4 and IL-13 can induce the activation of anti-inflammatory macrophages *in vitro*, which begin to appear gradually *in vivo* in the later stage of inflammation. They also have the ability to release an abundance of anti-inflammatory cytokines and trophic factors such as transforming growth factor beta (TGF-β) and Arginase-1 (ARG1), thus suppressing inflammation which leads to the tissue recovery process (4, 5). During the last few decades, these two types of macrophages have been classified as M1 macrophages and M2 macrophages. However, in recent years, with the development of macrophage phenotype studies and the discovery of different phenotypes of macrophages, people have realized that the activation of macrophages is a complex and systematic process. Therefore, some studies suggested macrophage polarization should not be simply described as M1 or M2 macrophages, but should be defined based on the macrophage activators such as LPS-induced macrophages, inflammatory macrophages, or M (LPS) (1, 6–8).

**Figure 1:**
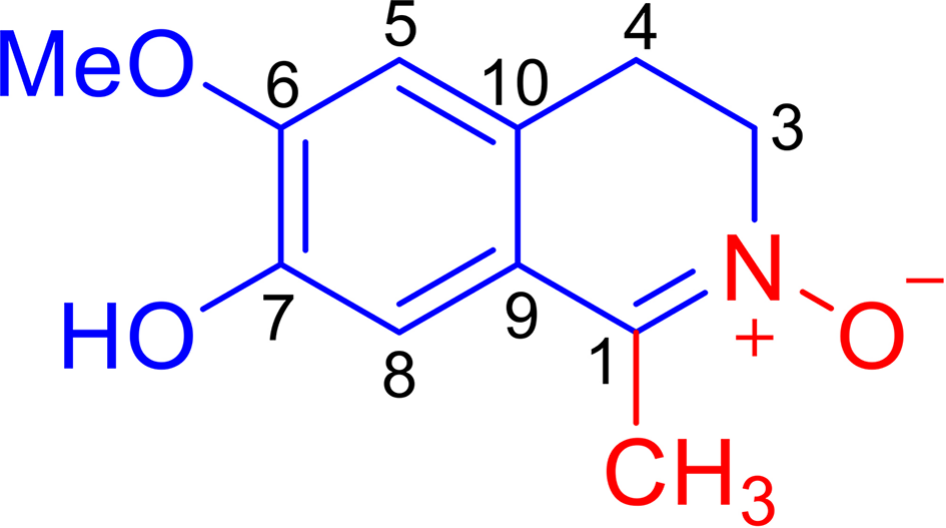
Structure of Litcubanine A (LA).

*Litsea cubeba* (Lauraceae), known as a Chinese medicinal plant, is widely distributed in eastern and southern China. Its fruits and roots, named “bi-cheng-qie” and “dou-chi-jiang”, respectively, are two important traditional Chinese medicines used for the treatment of rheumatic arthritis, atopic eczema, coronary heart disease, and cerebral apoplexy, which are closely related to inflammation (9–11). It has been reported that an extract of the *L. cubeba* root can attenuate adjuvant arthritis in rats and can relieve the swelling of ankles and joints in rats with collagen II-induced arthritis (12). Furthermore, an extract of *L. cubeba* bark inhibited the production of NO and prostaglandin E_2_ (PGE_2_) in LPS-induced RAW264.7 macrophages, whose over-expression can enhance inflammation and induce tissue destruction (13). Although a phytochemical investigation of *L. cubeba* revealed that some aporphine-type alkaloids and lignans are responsible for the weak inhibition of NO production in LPS-induced RAW264.7 macrophages (14), the anti-inflammatory constituents of *L. cubeba* and the underlying molecular mechanism involved has not been fully characterized. As a part of a program to systematically study the chemical diversity of natural anti-inflammatory products from *L. cubeba* (15–18), we discovered a novel isoquinoline alkaloid, Litcubanine A (LA) (**Figure 1**), which represents the first example of natural isoquinoline alkaloid possessing an unusual C-1 methyl and a N→O group. Our *in vitro* and *in vivo* studies indicate that LA possesses a significant anti-inflammatory activity by regulating the NF-κB signaling pathway in macrophages. Herein, we describe the isolation, structural properties, and the results of *in vitro* and *in vivo* studies characterizing the anti-inflammatory activity and mechanism of LA on macrophage regulation.

## Materials and Methods

### General Apparatus and Chemicals

All solvents used for extraction and chromatographic separation were of analytical grade. Optical rotations were measured using a Perkin-Elmer 343 automatic polarimeter. UV spectra were measured using a JASCO V-650 spectrometer. IR spectra were acquired using a Nicolet 5700 FT-IR spectrophotometer. Standard pulse sequences were used for all NMR experiments, which were run on a Bruker spectrometer (600 MHz for ^1^H or 150 MHz for ^13^C) equipped with an inverse detection probe. Residual solvent shifts for MeOD with TMS used as an internal standard. Accurate mass measurements were obtained using a Q-Trap LC/MS/MS (Turbo ionspray source) spectrometer. Column chromatography was performed using silica gel (200-300 mesh, Qingdao Marine Chemical Inc., China) and Sephadex LH-20 (Pharmacia Biotech AB, Uppsala, Sweden). HPLC separation was accomplished using HPLC components consisting of a Waters 1525 pump, a Waters 2487 dual λ absorbance, and a GRACE semipreparative (250 × 10 mm) column.

### Extraction and Isolation

For extraction and isolation of LA, please see **Supporting Information**.

### Data for LA

Amorphous red powder, UV (MeOH) *λ*_max_ (logϵ): 237 (3.61), 327 (3.19); IR (KBr) *ν*_max_ 3318, 2920, 2851, 1703, 1602, 1516, 1455, 1432, 1389, 1285, 1210, 1143, 1034, 854, 808 cm^-1^; ^1^H NMR (DMSO-*d*_6_, 600 MHz) and 13C NMR (DMSO-*d*_6_, 150 MHz) data, see **Table 1**; HRESIMS *m/z* 208.0967 [M + H]+ (calcd for C_11_H_14_NO_3_, 208.0968).

**Table 1:** NMR data (*δ*) for compound LA in MeOD*^a^*.

No.	LA	
	*δ*_H_ (mult, *J*, Hz)	*δ*_C_
1		138.9
2		
3	3.89 m	57.9
4	2.94 t (7.8)	26.6
5	6.87 s	111.4
6		147.5
7		145.3
8	6.80 s	111.5
9		122.7
10		122.5
Me-1	2.20 s	12.5
OMe	3.79 s	55.7

a NMR data (δ) were measured at 600 MHz for ^1^H NMR and at 150 MHz for ^13^C NMR. Proton coupling constants (J) in Hz are given in parentheses. The assignments were based on ^1^H-^1^H COSY, HSQC, and HMBC analyses.

### Chemicals and Antibodies

The *Porphyromonas gingivalis* derived LPS was from InvivoGen (San Diego, CA, USA). The iNOS, IκK, IκB, p65 NF-κB, phosphorylated IκK (p-IκK), p-IκB, and p-p65 NF-κB antibodies were from Cell Signalling Technology (Boston, MA, USA). The anti-β-actin antibody was from Sigma-Aldrich (St. Louis, MO, USA).

### Analytical Kits

The Mouse Peritoneal Macrophage Isolation Kit was Miltenyi Biotec (Bergisch Gladbach, Germany). The Mouse TNF-α ELISA Kit and IL-1beta ELISA Kit were eBioscience (San Diego, CA, USA). The NO Assay Kit and the iNOS Activity Assay Kit were Beyotime (Beijing, China).

### Isolation of Mouse Peritoneal Macrophages

Mouse peritoneal macrophages were isolated based on our previous research experience (1), and all experiments were performed under the institutionally approved protocols for the use of animal research (Capital Medical University#2012-x-53). Briefly, we gave six-to-eight-week-old male C57BL/6 mice an injection of 2 mL thioglycollate medium (4%) and waited for 5 days after injection, at which point the cells were collected by peritoneal lavage. Macrophages were then isolated using the Macrophage Isolation Kit by the depletion of non-target cells. By using a MACS Column in the magnetic field of a MACS Separator, we depleted the magnetically labelled cells by retaining them inside the column, and collected the unlabelled macrophages which passed through the column. To characterize the classification of isolated cells, we used flow cytometric analysis with an anti-F4/80-FITC antibody and an anti-MHC ClassII(I-ab)-PE antibody. The results showed that >98% of the isolated cells were macrophages. The macrophages were cultured in the RPMI medium (Invitrogen, Carlsbad, CA, USA), with 15% heat-inactivated fetal bovine serum (FBS; Equitech-Bio, Kerrville, TX, USA), 5% pen/Strep (Invitrogen), and 5% glutamine (Invitrogen). Macrophages were cultured at 37°C, with 5% CO_2_, in a humidified atmosphere. All the isolated cells were induced no more than one week after isolation.

### Culture of RAW 264.7 Macrophages

RAW264.7 macrophages were purchased from China Infrastructure of Cell Line Resources (Beijing, China), and were cultured in Dulbecco’s modified Eagle’s medium (DMEM, Invitrogen) with 10% FBS, 2 mM L-glutamine (Invitrogen), 100 U/ml penicillin, and 100 μg/mL streptomycin (Invitrogen). The cells were cultured at 37°C, with 5% CO_2_, in a humidified atmosphere. Every 2-3 days, the cell medium was changed. When the cells became 70% to 80% confluent, they would be passaged.

### Induction of Macrophages by LPS

Based on our macrophage research experience (1) and other previous studies (19, 20), we treated RAW264.7 macrophages with 1 μg/mL LPS for 24 h to induce the expression of inflammatory factors. We determined the *iNOS* expression levels using quantitative real time PCR, Western blot, and ELISA analyses, and evaluated TNF-α and IL-1β expression levels with ELISAs. The ratio of iNOS positive cell numbers/total cells were evaluated with immunohistochemistry staining. The activation of iNOS protein was evaluated with an iNOS Activity Assay Kit and NO levels were determined with a NO Assay Kit.

### LA Treatment Inhibits the Expression of LPS-Induced Inflammatory Factors in Macrophages

RAW264.7 macrophages and mouse peritoneal macrophages were seeded at a density of 2 × 10^5^ cells/well in 6-well plates after isolation, and were cultured at 37°C in a 5% CO_2_ incubator. In addition to LPS inducement, the macrophages were treated with different concentrations of LA (0 nM, 10 nM, 100 nM, 1 μM, 10 μM) for 24 h. The negative controls were cells treated with phosphate buffered saline (PBS) or DMSO. 100 nM Dex was used as the positive control (21). After that, the inflammatory macrophage activation levels in different groups were determined as previously described (1).

### Quantitative Real-Time PCR Analysis

After treatment with LPS and LA, total RNA was extracted from each group with Trizol reagent (Invitrogen). cDNAs were synthesized according to the manufacturer’s protocol. Real-time PCR reactions were applied using the Power SYBR^®^ Green PCR Master Mix (Life Technologies, Warrington, UK) with *iNOS, TNF-α, IL-1β*, and *GAPDH* primers (**Table S1** in **Supporting Information**).

### ELISA Analysis

To evaluate the expression levels of TNF-α and IL-1β, we collected cell culture supernatants and performed analysis with corresponding ELISA kits according to the manufacturer’s instructions.

### Western Blot Analysis

Western blot was performed based on our previous research experience (1). Total protein was extracted with NE-PER nuclear and cytoplasmic extraction reagents (Thermo, Boston, MA, USA). Fifty μg aliquots of proteins were separated on 10% polyacrylamide-SDS gels (Pplygen, Beijing, China) and transferred to ImmobilonTM-P membranes (Millipore, Birrica, MA, USA). After blocking with TBS/5% non-fat dry milk (Pplygen) for 1 h at room temperature, the membranes were incubated with primary antibodies overnight at 4°C. The next day, the membranes were washed and incubated with horseradish peroxidase-conjugated secondary antibodies (Pierce, Malibu, CA, USA) for 1 h at room temperature. Antibody binding was visualized using an enhanced chemiluminescence kit, according to the manufacturer’s protocols (Pierce).

### Immunohistochemistry

For the *in vitro* study, RAW264.7 macrophages and primary mouse peritoneal macrophages were seeded into 12-well plates which contained glass coverslips at a density of 2 × 10^5^ cells/well. The LA group was treated with 100 nM LA and 1 μg/mL LPS for 24 h. The LPS group was treated with DMSO and 1 μg/mL LPS for 24 h, and the control group was treated with DMSO for 24 h. After that, all glass coverslips were harvested and fixed in 4% paraformaldehyde (PFA). The sections were incubated with 3% hydrogen peroxide for 10 min at room temperature, then blocked with 10% serum for another 60 min at 37°C to reduce non-specific staining. The glass coverslips were then incubated with primary antibodies (50 μg/mL) at 4°C overnight. The next day, all pre-treated sections were washed and incubated with biotinylated secondary antibodies (1 μg/mL) for another 1 h at room temperature. Finally, horseradish peroxidase complexes were visualized using a diaminobenzidine substrate. The counterstained slides were observed using a light microscope (OLYMPUS, Tokyo, Japan), and the numbers of positively stained cells in at least five random fields were calculated and analyzed with semi-quantification.

For the *in vivo* study, the sections (5 μm thick) were cut and mounted onto slides, and were stained according to the manufacturer’s protocol. All sections were incubated with 3% hydrogen peroxide for 10 min, and blocked with 10% serum for another 1 h at 37°C to reduce the non-specific staining. After that, the sections were incubated with iNOS antibody (Cell Signaling Technology, Boston, MA, USA) at 4°C overnight. Biotinylated secondary antibodies were added and incubated at room temperature for 1 h, and finally, the horseradish peroxidase complex was added with the diaminobenzidine substrate for visualization.

### Flow Cytometric Analysis

The number of isolated peritoneal macrophages were determined by flow cytometric analysis. The cells were fixed with 4% PFA, then incubated with 5% normal serum for blocking. The cells were then incubated for 30 min with the anti-F4/80-FITC antibody and anti-MHC ClassII(I-ab)-PE antibody in the dark. After that, cell samples were immediately analyzed by flow cytometry (FACSCalibur, BD Bioscience).

To analyze total cell apoptosis, the induced cells were incubated with different concentrations of LA (0 nM, 10 nM, 100 nM, 1 μM, 10 μM) for 24 h. Cells were then harvested by trypsin digestion without EDTA, fixed with 4% PFA and incubated with 5% normal serum for blocking. Apoptosis was assessed using an Annexin V Apoptosis Detection Kit FITC (Bioscience, San Diego, CA, USA) according to the manufacturer’s instructions using a Calibur flow cytometer (BD Immunocytometry Systems, San Jose, CA, USA). A minimum of three independent experiments were performed.

### Cell Proliferation After Treatment With LA

To analyze cell proliferation after treatment with LA, the cells were treated with different concentrations of LA (0 nM, 10 nM, 100 nM, 1 μM, 10 μM) for 24 h. A Cell Counting Kit (CCK)-8 (Dojindo Laboratories, Kumamoto, Japan) was used according to the manufacturer’s instructions. Ten μLs of CCK-8 solution were added to each well and incubated for 1 h, and OD values were read at 450 nm. All experiments were repeated at least three independent times.

### Larva Manipulation for Inflammation Assays, Imaging, and PCR

All *in vivo* zebrafish experiments described in this study were performed under the requirements of the Association for Assessment and Accreditation of Laboratory Animal Care (AAALAC) international certification. The license number of the experimental animal is SYXK (Zhejiang) 2012-0171, and the ethic statement number was IACUC 001485. Caudal fin amputation was applied on larvae that were 3 days post-fertilization (dpf) as previous studies described (22). Briefly, the caudal fin of each zebrafish was amputated using a sterile scalpel, posterior to the muscle and notochord under anesthesia with 0.016% Tricaine (ethyl 3-aminobenzoate, Sigma Aldrich, Birrica, MA, USA). Different doses of LA or Dex were injected using a microinjector IM-300 (Narishige, Tokyo, Japan) into the larvae in different groups (10 embryos in each group) after the amputation. Macrophage tracking was performed as previously described (23) using a confocal TCS SP5 inverted microscope accompanied by HCXPL APO 40×/1.25–0.75 oil objective (Leica, Wetzlar, Germany).

For further assays, larvae samples were crushed to collect the cell sample with a 70-μm cell strainer. Then, the cells were washed with PBS/2 mM ethylenediaminetetraacetic acid (EDTA)/2% fetal calf serum (FCS), and the sample was filtered through another 40-μm cell strainer (23). After that, PCR analysis was performed as described above.

### Molecular Docking Simulations

The structural coordinates of LA were obtained from the human metabolome database (www.HMDB.ca). Discovery Studio 2016 was used to convert data to the PDB file format. The structural coordinates of iNOS, TNF-α, and IL-1β were retrieved in PDB format from the RCSB protein data bank (www.rcsb.org: PDB ID: 3E6N, 3BRV and 3EBD, respectively), according to the previous studies (24, 25).

### Wound-Healing Mouse Model

Six-week-old male C57BL/6 J mice were purchased from Vital River (Beijing, China) and housed in separate pathogen-free animal facilities under controlled temperature (25°C) and photoperiods (12:12-h light: dark cycle) and fed a standard diet and tap water. This study was conducted following the approved guidelines established by the Animal Ethics Committee of the School of Stomatology, Capital Medical University (Beijing, China), and conformed to the ARRIVE Guidelines. All animal experiments were performed under the institutionally approved protocols for the use of animals in research (Capital Medical University #2012-x-53).

The animals were anesthetized with 1% pentobarbital, then were subjected to standardized skin wounds as previously described (26). A 5-mm-diameter full-thickness excisional wound was performed on the shaved dorsal skin of the mice using a biopsy punch. After the surgery, mice in the Litcubanine A group were injected with 1 μM LA solution into the wound tissue for 3 days, and the mice in the control group were injected with the same amount of PBS in approximately the same area (five animals for each group). The mice were sacrificed after 3 days, and the defect area was observed and assessed using ImageJ. The samples were then fixed with 4% PFA, and embedded in paraffin. Sections were deparaffinized and stained with immunohistochemistry staining. To minimise potential confounders, the researcher who performed the surgery and the researcher who responsible for analysis were not the same one, and the researcher who responsible for analysis was not aware of the groups of the samples.

### Statistical Analysis

All statistical analyses were performed using SPSS13.0 software. All data points are shown as means ± standard deviation (SD). IC_50_ values (concentration of inhibitor that reduces enzyme activity by 50%) were evaluated by nonlinear regression in GraphPad Prism 6.0 software (GraphPad Software, Inc., La Jolla, CA, USA). Student’s t-test was used to compare two sets of data that were normally distributed, based on normality plots and tests. We checked equal variance and performed multiple-variable comparisons with one-way analysis of variance (ANOVA). Student Newman-Keuls test was used to analyze the two-by-two comparisons between means. All statistical analyses were performed with at least three biological replicates.

## Results

### Isolation and Structural Characterization

An EtOH extract of the twigs of *L. cubeba* was concentrated *in vacuo*, after which the residue was resuspended in H_2_O and then partitioned with EtOAc. The aqueous phase was then subjected to column chromatography (CC) over HP-20 macroporous adsorbent resin, followed by HPLC purification to obtain Litcubanine A.

**Figure 2:**
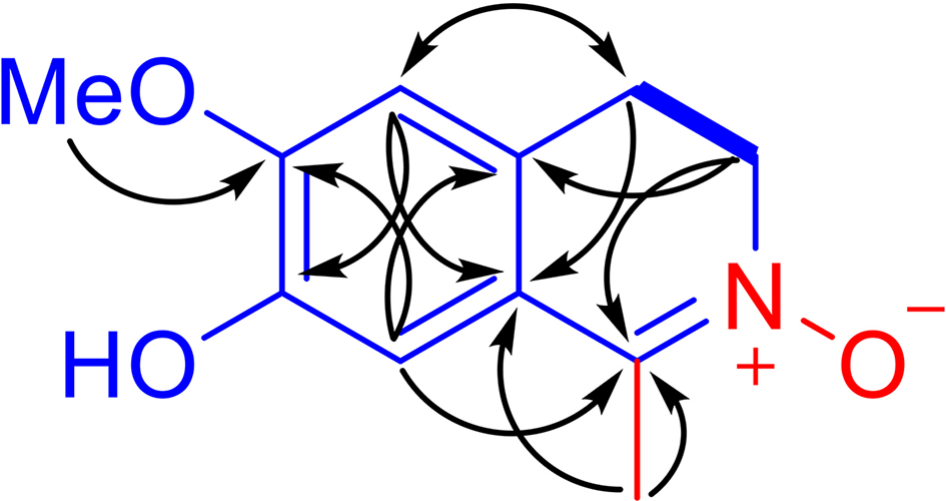
Main ^1^H-^1^H COSY (bold lines) and selected HMBC correlations (arrows, from ^1^H to ^13^C) of LA.

LA was obtained as an amorphous red powder with a molecular formula of C_11_H_13_NO_3_, assigned by the positive HRESIMS data. These data also indicated that LA contains 6 double bond equivalents, which, based on the NMR data (**Table 1**), were initially attributed to one aromatic ring, one C=N double bond, and one ring. The IR spectrum was consistent with the presence of a hydroxy group (3318 cm^-1^) and an aromatic moiety (1602, 1516, and 1455 cm^-1^), supporting some of the above assignments that were made from analysis of the NMR data. The presence of a 1,2,4,5-tetrasubstituted aromatic ring was indicated from the ^1^H NMR data, which exhibited two aromatic singlet protons at *δ*_H_ 6.87 (1H, s, H-5) and 6.80 (1H, s, H-8). Furthermore, two methylene moieties at *δ*_H_ 3.89 (2H, m, H_2_-3), 2.94 (2H, t, *J* = 7.8 Hz, H_2_-4), one methoxy group at *δ*_H_ 3.79 (3H, s, OMe-6) and a methyl singlet at *δ*_H_ 2.20 (3H, s, Me-1) were present in the ^1^H NMR spectrum. In a consistent manner, the ^13^C NMR spectrum showed a total of 11 carbon signals assigned as 6 aromatic carbons, one methyl, two methylenes, one methoxy group, and one C=N moiety assessed with the aid of a HSQC experiment. ^1^H -^1^H COSY correlations of H_2_-3 with H_2_-4 and HMBC correlations from Me-1 to C-1 and C-9, from H_2_-3 to C-1 and C-10, from H_2_-4 to C-5 and C-9, from H-5 to C-4, C-7 and C-9, from H-8 to C-1, C-6 and C-10, and from OMe to C-6 (**Figure 2**). Together, with their chemical shift values, this information preliminarily established LA as 7-hydroxy-6-methoxy-1-methyl-3,4-dihydroisoquinoline. Although this structure contained all the necessary 11 carbons, 2 oxygens and 1 nitrogen, one more oxygen was required by the molecular formula. Clearly, the presence of an N→O group in LA was suggested by the distinctive chemical shift of C-1 (*δ*_C_ 138.9) and C-3 (*δ*_C_ 57.9) compared with isoquinoline alkaloids with a similar C=N group (27). On the basis of these data, the structure of LA was determined as 7-hydroxy-6-methoxy-1-methyl-3,4-dihydro isoquinoline-^15^ *N*-oxide.

### Anti-LPS-Induced NO Production Assay

Taking into account that *L. cubeba* is widely used for the treatment of inflammatory diseases, LA was assessed for its potential anti-inflammatory activity against LPS-induced production of NO in RAW264.7 macrophages. The production of NO was determined using a NO Assay Kit (Beyotime, Beijing, China). The results showed that LA effectively inhibited the LPS-induced up-regulation of NO (IC_50_ 300.9 nM), and was not significantly different when compared to the positive control, dexamethasone (Dex, IC_50_ 269 nM), demonstrating the significant anti-inflammatory activity of LA (**Figure S1** in **Supporting Information**).

### Mechanism of Inhibition of LA Against NO Production *In Vitro*

A key mediator of macrophages in immune activation and inflammation is iNOS, which is upregulated by inflammatory stimulation and produces NO from L-arginine. To explore the mechanism of inhibition of LA against NO production *in vitro*, we first detected the iNOS expression levels in LA-treated RAW264.7 macrophages and determined whether LA inhibited NO production through the iNOS-related pathway.

**Figure 3:**
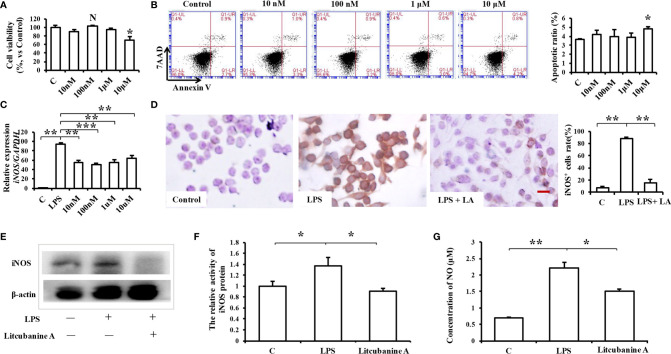
Treatment with 100 nM LA inhibits the LPS-induced expression of iNOS in RAW264.7 macrophages *in vitro*. **(A)** CCK8 analysis showing that treatment with 100 nM LA had no effect on cell proliferation (n=3). **(B)** Flow cytometry analysis showing that treatment with 100 nM LA had no effect on cell apoptosis (n=3). **(C)** PCR analysis showing that treatment with 100 nM LA significantly decreased the LPS-induced expression of *iNOS* in RAW264.7 macrophages. Other concentrations of LA also downregulated the expression levels of *iNOS* induced by LPS but 100 nM downregulated the expression level of *iNOS* more stably compared to the other concentrations (n=3). **(D)** Immunocytochemical staining assays showing that the LPS-induced iNOS-positive cell ratio was significantly increased after LPS treatment (88.7% ± 1.34%) compared to the control group (7.33% ± 2.50%), and that treatment with 100 nM LA decreased the LPS-induced iNOS positive cell ratio (15.66% ± 5.07%) (n=5). **(E)** Western blot results confirming that treatment with 100 nM LA decreased the protein expression level of total iNOS in RAW264.7 macrophages. **(F)** Treatment with 100 nM LA inhibited the up-regulation of iNOS protein activity induced by LPS in RAW264.7 macrophages (n=3). **(G)** Treatment with 100 nM LA decreased the expression level of the downstream product NO induced by LPS in RAW264.7 macrophages (n=3). All results are representative of at least three independent experiments/samples. Results are expressed as means ± standard deviation (SD), and statistical significance is shown as: N P > 0.05, *P < 0.05, **P < 0.01, ***P < 0.001.

To determine a suitable concentration of LA to use *in vitro*, we treated RAW264.7 macrophages with different doses of LA (10 nM, 100 nM, 1 μM, 10 μM) and detected its cytotoxicity using CCK8 assays and flow cytometric analysis. The results indicated that treatment with LA below 10 μM concentration had no cytotoxicity, but the 10 μM LA treatment inhibited cell proliferation (**Figure 3A**) and induced cell apoptosis (**Figure 3B**). Moreover, we found that all doses of LA significantly decreased the LPS-induced expression of *iNOS*. However, the downregulation of *iNOS* was most significant at the 100 nM concentration of LA (**Figure 3C**; *P* < 0.01). Therefore, in subsequent assays, we used 100 nM LA to treat macrophages *in vitro*. Immunocytochemical staining showed that the percentage of LPS-induced iNOS-positive RAW264.7 macrophages significantly increased after LPS stimulation (88.7% ± 1.34%) compared to the control group (7.33% ± 2.50%) (**Figure 3D**). However, 100 nM LA treatment reduced the percentage of LPS-induced iNOS-positive macrophages (15.66% ± 5.07%). Moreover, western blot analysis confirmed the inhibitory effect of LA on the protein expression level of iNOS in RAW264.7 macrophages (**Figure 3E**).

We then investigated the reason as to why LA significantly inhibited the LPS-induced up-regulation of iNOS in macrophages by detecting iNOS protein activity using an iNOS Activity Assay Kit (Beyotime, Beijing, China). When we detected iNOS activity in RAW264.7, we found that treatment with 100 nM LA inhibited the up-regulation of LPS-induced iNOS activity (**Figure 3F**), which indicates that LA can directly inhibit iNOS protein activity. To further verify those results, the expression of the downstream product NO was also investigated. The results showed that treatment with LA effectively inhibited the LPS-induced up-regulation of NO (**Figure 3G**).

**Figure 4:**
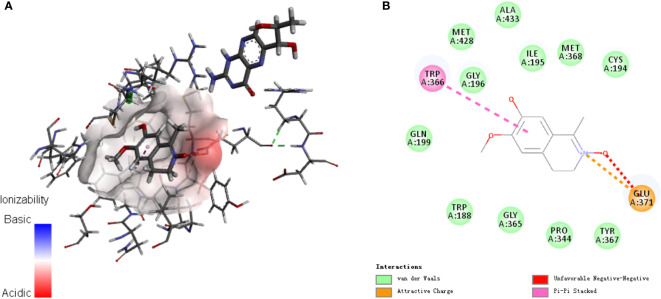
Analysis of **(A)** active targets in the structure of LA and **(B)** simulation docking with the iNOS protein crystal structure. The results demonstrate that in theory, LA can directly bind the iNOS protein *via* interactions with TRP 366 and GLU371 residues for its inhibitory effect (23).

Analysis of the active targets in the structure of LA and simulation docking with the iNOS protein crystal structure proved that in theory, LA can directly bind the iNOS protein *via* interactions with TRP 366 and GLU371 residues to produce its inhibitory effect (**Figures 4A, B**) (24).

### The Anti-Inflammatory Activity of LA in Mouse Peritoneal Macrophages *In Vitro*

**Figure 5:**
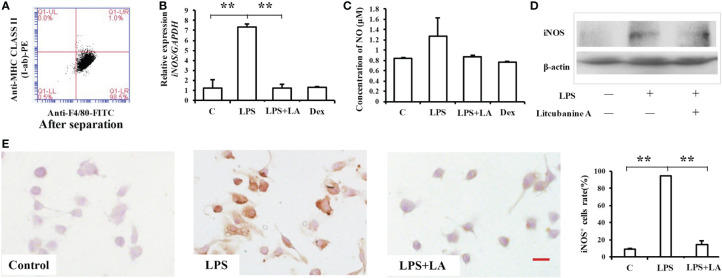
Treatment with 100 nM LA inhibits the expression of LPS-induced iNOS in primary macrophages **(A)** Flow cytometric analysis showing that >98% of cells were macrophages after separation. **(B)** Real-time PCR analysis showing that treatment with LA decreases *iNOS* expression induced by LPS in peritoneal macrophages, and that the inhibitory effect was similar to that elicited by Dex (n=3). **(C)** LPS-induced NO expression was also inhibited by treatment with LA, which showed no significant difference compared with Dex (n=3). **(D)** Western blot analysis confirms that treatment with 100 nM LA decreases the protein expression level of total iNOS in peritoneal macrophages. **(E)** Immunocytochemical staining showing that the LPS-induced iNOS positive cell ratio significantly increased after LPS inducement (94.24% ± 0.60%) compared to the control group (9.35% ± 0.10%). However, treatment with 100 nM LA reduced the percentage of LPS-induced iNOS positive macrophages (14.36% ± 4.01%) (n=5). Scale bar = 20 μm. All results are representative of at least three independent experiments/samples. Results are expressed as means ± standard deviation (SD), and statistical significance is shown as **P < 0.01.

RAW264.7 macrophages are a kind of pathological macrophage, and the results obtained using RAW264.7 may not be representative of normal macrophages. Therefore, some studies have suggested that it is necessary to verify the *in vitro* results obtained with RAW264.7 macrophages using primary macrophages (1). Thus, in this study, we isolated primary mouse peritoneal macrophages and identified them using flow cytometry. The results showed that >98% of the cells were macrophages (**Figure 5A**). The *in vitro* results reported above were then confirmed using these primary peritoneal macrophages. Analysis of *iNOS* and NO expression confirmed that 100 nM LA inhibited the LPS-induced inflammatory effect in these primary peritoneal macrophages, and moreover, there was no significant difference in the inhibitory effect between LA and the positive control Dex at the same concentration (**Figures 5B, C**). Western blot (**Figure 5D**) and immunocytochemical staining (**Figure 5E**) showed identical results to those obtained using RAW264.7 macrophages. The percentage of LPS-induced iNOS positive macrophages was significantly increased after LPS stimulation (94.24% ± 0.60%) compared to the control group (9.35% ± 0.10%). Furthermore, treatment with 100 nM LA significantly reduced the percentage of LPS-induced iNOS positive macrophages (14.36% ± 4.01%). Thus, our data shows that 100 nM LA stably inhibited LPS-induced iNOS expression *in vitro*, both in RAW264.7 macrophages and in primary mouse peritoneal macrophages.

### LA Inhibits Inflammatory Macrophage Activation *via* the NF-κB Pathway

**Figure 6:**
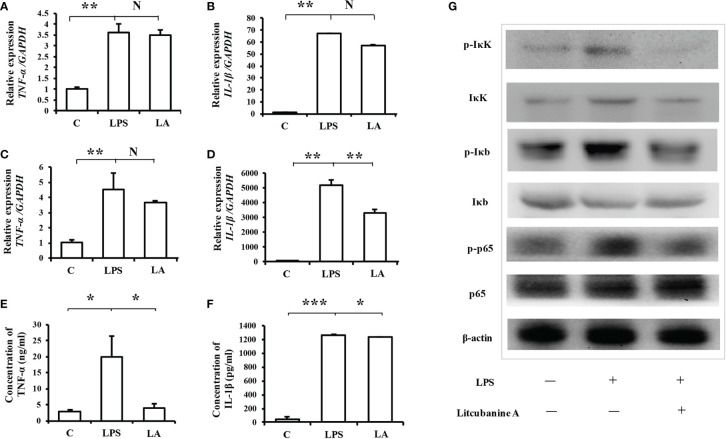
Treatment with LA inhibits the expression of inflammatory factors induced by LPS *via* the NF-κB pathway **(A, B)** Real-time PCR analysis showing that in peritoneal macrophages, the up-regulation of *TNF-α* and *IL-1β* after LPS treatment can be inhibited by treatment with 100 nM LA (n=3). **(C, D)** Treatment with LA also inhibited *TNF-α* and *IL-1β* mRNA expression levels in RAW264.7 macrophages (n=3). **(E, F)** ELISA results confirming that LPS treatment promotes the expression level of TNF-α and IL-1β, which is decreased by treatment with LA in RAW264.7 macrophages (n=3). **(G)** Western blot showing that LPS treatment increases the protein expression levels of p-IκK, p-Iκb and p-p65. In contrast, treatment with LA inhibits the upregulation of those proteins induced by LPS. All results are representative of at least three independent experiments/samples. Results are expressed as means ± standard deviation (SD), and statistical significance is shown as N P > 0.05, *P < 0.05, **P < 0.01, ***P < 0.001.

**Figure 7:**
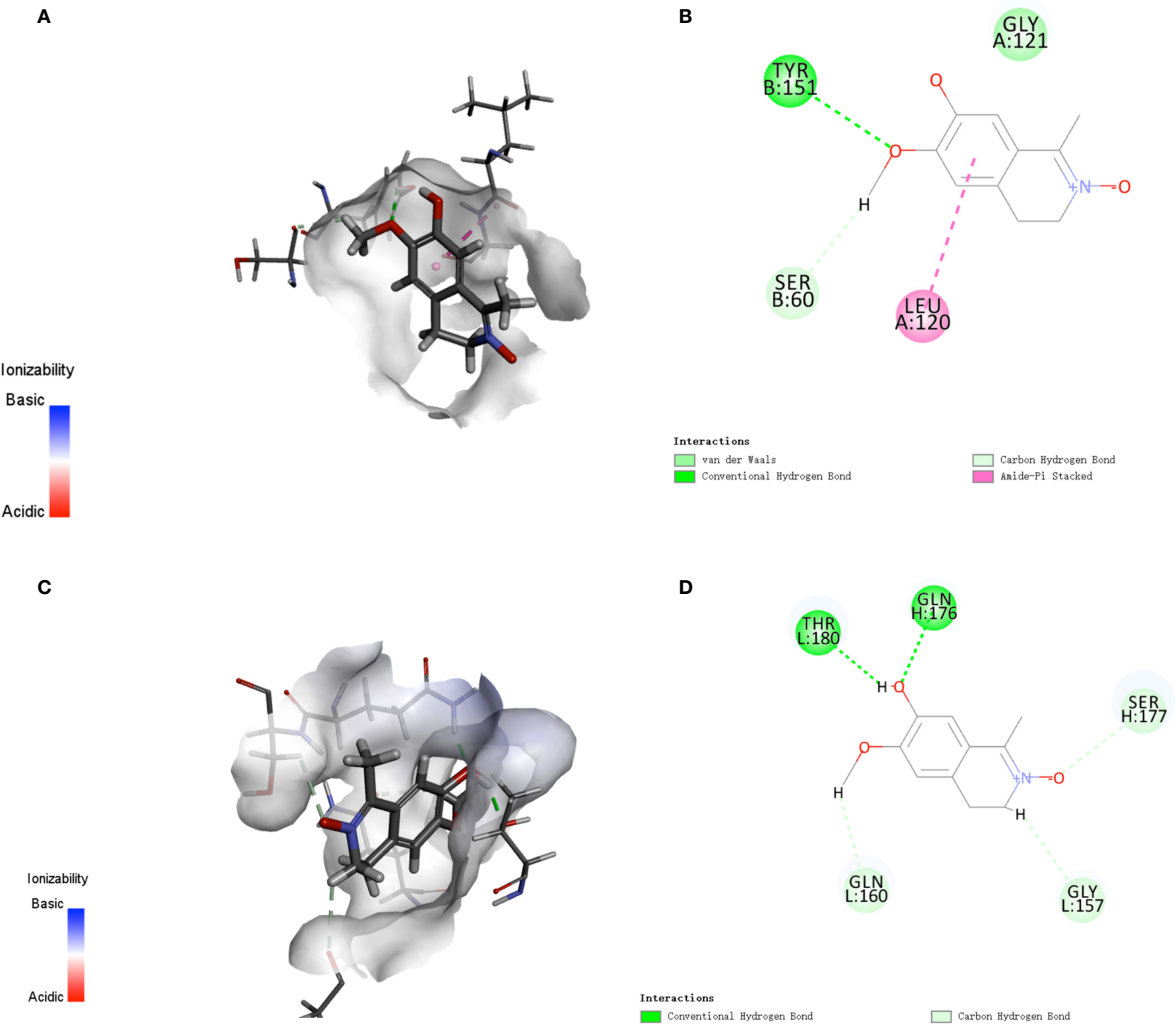
Analysis of active targets in the structure of LA and simulation docking with TNF-α and IL-1β **(A, B)** Analysis of active targets in the structure of LA and simulation docking with the TNF-α crystal structure shows that in theory LA can directly bind to TNF-α *via* an interaction with the LEU 120 and TYR 151 residues to elicit its inhibitory effect (24). **(C, D)** Analysis of the active targets in LA and simulation docking with the IL-1β crystal structure shows that in theory LA can directly bind to IL-1β *via* interactions with the THR180 and GLN176 residues to elicit its inhibitory effect.

In addition, since iNOS is considered one of the important markers of the inflammatory macrophages, we hypothesized that LA might also play a regulatory role during the activation of inflammatory macrophages. Therefore, we then used real-time PCR analysis to detect other inflammatory factor’s expression levels including *TNF-α* and *IL-1β*, which could also be markers of the inflammatory macrophages. The results showed that 100 nM LA inhibited the LPS-induced expressions of *TNF-α* and *IL-1β* in peritoneal macrophages (**Figures 6A, B**) and in RAW264.7 (**Figures 6C, D**). We also investigated the expression of TNF-α and IL-1β using ELISA analysis. The ELISA results confirmed that 100 nM LA inhibited the expression of these two factors in RAW264.7 (**Figures 6E, F**). These data suggested that LA could inhibit LPS-induced activation of macrophages. Next, we investigated a pathway that might have contributed to the LA inhibition of inflammatory macrophage activation using RAW264.7. Previous studies reported that the NF-κB pathway plays a key role during the LPS-induced macrophage activation (1). Therefore, we investigated the expression levels of IκK, p-IκK, IκB, p-IκB, p65 NF-κB, and p-p65 NF-κB proteins. We found that LPS treatment increased the expression of p-IκK, p-IκB, and p-p65 NF-κB proteins, and that LA inhibited this upregulation (**Figure 6G**). These data suggested that LA inhibited the activation of LPS-induced inflammatory macrophages that expressed the inflammatory factors. Moreover, analysis of active targets in the structure of LA and simulation docking with TNF-α and IL-1β showed that in theory, LA can directly bind to those inflammatory factors resulting in the inhibitory effect (**Figures 7A–D**) (25).

**Figure 8:**
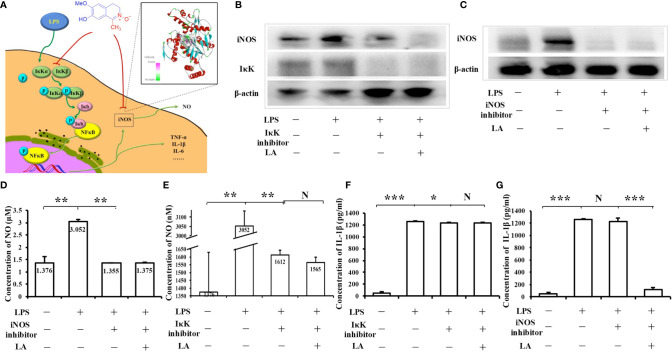
The anti-inflammatory effect of LA is achieved by inhibiting both the NF-κB pathway and the activity of iNOS protein **(A)** Scheme based on the results of this study showing that LA inhibits the LPS-induced macrophages by both the NF-κB pathway and the downstream product iNOS protein. **(B)** Western blot showing that an IκK inhibitor effectively suppresses the expression level of IκK in RAW264.7 macrophages. Moreover, when the NF-κB pathway was blocked with an IκK inhibitor, the expression of downstream iNOS protein induced by LPS was decreased. However, the expression of iNOS could be further inhibited by treatment with LPS + LA compared to LPS only, proving that LA can specifically inhibit iNOS protein. **(C)** Western blot showing that an iNOS inhibitor effectively suppressed the expression level of iNOS in RAW264.7 macrophages. **(D)** NO levels were significantly reduced after treatment with an iNOS inhibitor, which was not affected by treatment with additional LA under such conditions (n=3). **(E)** When the NF-κB pathway was blocked with an IκK inhibitor, NO production was inhibited and could be further decreased by treatment with additional LA (n=3). **(F)** ELISA analysis showing that when the upstream NF-κB pathway was blocked with an IκK inhibitor, the expression of IL-1β induced by LPS was decreased, and this was not affected by further treatment with LA (n=3). **(G)** When iNOS activity was blocked by an iNOS inhibitor, the expression of IL-1β remained stable, but the additional treatment with LA significantly inhibited the expression of IL-1β induced by LPS (n=3). All results are representative of at least three independent experiments/samples. Results are expressed as means ± standard deviation (SD), and statistical significance is shown as N P > 0.05, **P < 0.01, ***P < 0.001.

Taken together, LA inhibited the IκK/IκB/NF-κB pathway, thus inhibiting the activation of inflammatory macrophages, leading to the down-regulation of inflammatory factors including iNOS, TNF-α, and IL-1β. Moreover, LA could further directly bind to the iNOS protein and inhibit its activity so that it decreased the expression of NO in macrophages (**Figure 8A**). To verify these molecular mechanisms, we used an IκK inhibitor (S2864, Selleck, Houston, TX, USA) and an iNOS inhibitor (S8337, Selleck) to block those two pathways, and used a western blot to confirm the effects of those two inhibitors (**Figures 8B, C**). Interestingly, the western blot results showed that when the NF-κB pathway was blocked with the IκK inhibitor, the expression of iNOS induced by LPS was decreased. However, the expression of iNOS could be further inhibited by treatment with LPS + LA, compared with the LPS intervention only (**Figure 8B**), proving that LA can specifically inhibit iNOS, and this result is consistent with our hypothesis. In order to further clarify the conclusion, the level of the downstream product NO was also detected. The results showed that when the activity of iNOS protein was blocked, the level of NO was significantly reduced, and could not be influenced by the additional LA treatment under such conditions (**Figure 8D**). However, if the NF-κB pathway was blocked with the IκK inhibitor, the expression of NO was inhibited, and could be further decreased by additional treatment with LA (**Figure 8E**). This result proved again that LA can directly inhibit iNOS activity.

To verify the other target pathway, NF-κB, we chose IL-1β, another downstream inflammatory factor, as the indicator. IL-1β is secreted by activated an NF-κB pathway. The results of ELISA analysis showed that when upstream of the NF-κB pathway was blocked with an IκK inhibitor, the expression of IL-1β induced by LPS was decreased, which could not be affected by further treatment with LA (**Figure 8F**). However, if the iNOS activity was blocked by the iNOS inhibitor, the expression of IL-1β remained stable since the upstream NF-κB pathway was still activated by LPS, but the additional LA treatment significantly inhibited the expression of IL-1β induced by LPS (**Figure 8G**). This is the convincing proof that LA decreases IL-1β expression by inhibiting the upstream NF-κB pathway. These data indicate that the anti-inflammatory effect of LA is achieved by inhibiting both the NF-κB pathway and the activity of iNOS proteins in macrophages.

### *In Vivo* Anti-Inflammatory Activity of LA

**Figure 9:**
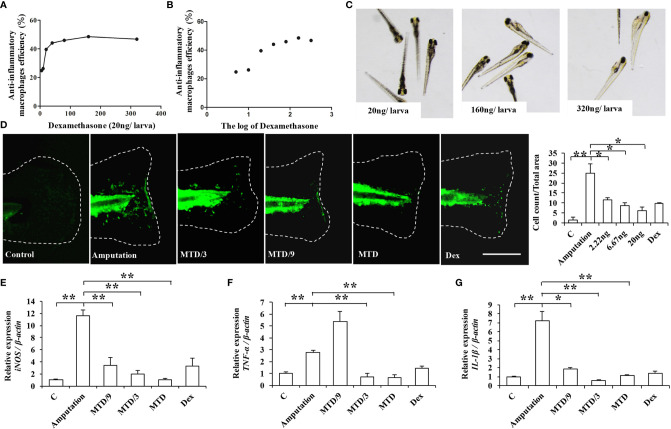
LA inhibits the recruitment of macrophages to the inflammation site and inhibits the secretion of local inflammatory factors at the early stage of inflammation in the caudal fin resection model in zebrafish larvae **(A, B)** Graphs showing the effects of Dex. The 20 ng/larva injection dose of Dex shows a significant anti-inflammatory effect, with anti-inflammatory effects on macrophages up to 40% (P < 0.05). **(C)** The 20 ng/larva injection dose of Dex had no effect on the physiological state of zebrafish, while the higher injection doses caused a loss of balance and even pericardial edema. **(D)** Macrophages were recruited to the wounds in amputated larvae at 24 hpA, while an injection of LA significantly inhibited the cell count of macrophages in the wound tissues, which was most significantly shown in the MTD group (20.0 ng/larva) (n=10). **(E)** Real-time PCR results show that an LA injection inhibited the expression levels of *iNOS* in wound tissues, and 20.0 ng/larva LA downregulated the expression level of *iNOS* more than the other doses (n=3). **(F)** 20.0 ng/larva LA decreased the expression of *TNF-α* in the wound tissues, which was not significantly different compared with the positive control group Dex (n=3). **(G)** LA also significantly decreased the expression of *IL-1β*, with an inhibitory effect stronger than that of Dex (n=3). Scale bar in **(D)** = 100 μm. Results are expressed as means ± standard deviation (SD), and statistical significance is shown as N P > 0.05, *P < 0.05, **P < 0.01.

After identifying the anti-inflammatory activity and the underlying molecular mechanism of LA, we further investigated the immunomodulatory functions of LA *in vivo* using a caudal fin resection model in zebrafish larvae, which allows the *in vivo* monitoring of macrophage migration at the site of an injury (23). First, we determined the maximum tolerated dose (MTD) of LA administered to zebrafish larvae by giving different injection doses of LA to zebrafish and observing their physiological state (28). The results showed that an injection dose of 40 ng per larva caused the zebrafish to roll over and lose their balance, while an injection dose of 20 ng per larva had no effect on the physiological state of zebrafish. Therefore, we chose 20 ng per larva as the MTD for LA for the *in vivo* experiment. The caudal fins of Tg (coroa1: EGFP) zebrafish larvae were resected, and they were given injection doses of 1/9 MTD, 1/3 MTD and MTD as experimental groups while the negative control group was injected with DMSO. 20 ng per larva of Dex was used as the positive control group based on the LA injection dose and the results of the Dex effect curve (**Figures 9A–C**). The relative macrophage cell counts at the injury sites were observed at 24 hours post-amputation (hpA) according to the previous study (29). We found that macrophages were recruited to the wounds in amputated larvae at 24 hpA, while an injection of LA significantly inhibited the cell count of macrophages in the wound tissues, which showed a stronger effect than the positive control group (**Figure 9D**). Moreover, the results showed a direct correlation with the inhibitory effects and the dose of LA injected, and the downregulation effect on macrophages was the most significant in the MTD group (20.0 ng/larva, **Figure 9D**). Further, real-time PCR was used to analyze the mRNA expression levels of *iNOS, TNF-α,* and *IL-1β* in the wound tissues, and the results confirmed that the LA injection inhibited the up-regulation of those inflammatory factors in amputated larvae 24 hpA *in vivo* (**Figures 9E–G**). These data indicated that LA inhibits the recruitment of macrophages to the inflammation site and inhibits the secretion of local inflammatory factors at the early stage of inflammation *in vivo*.

**Figure 10:**
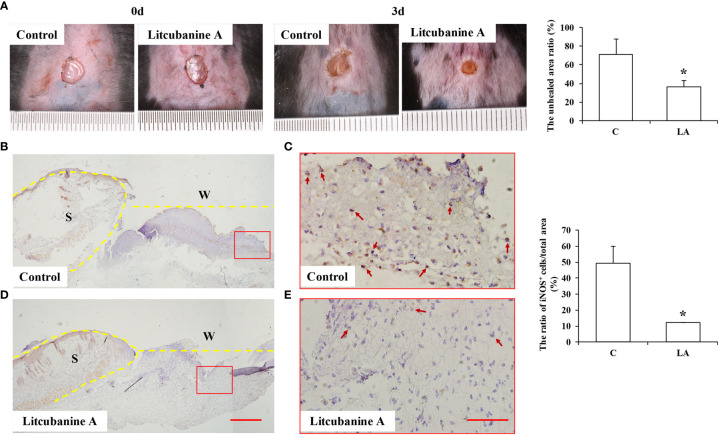
LA injection promoted wound healing by inhibiting the local inflammatory macrophages activation **(A)** LA injection significantly promoted wound healing compared to the control group (n=5). **(B–E)** The local iNOS^+^ inflammatory macrophages were observed using immunohistochemistry staining, and the results showed that by the third day after the surgery, the wound tissues in the control group showed an overexpression of iNOS (49.18% ± 10.84%). However, the iNOS^+^ cells ratio was lower in LA group (12.22% ± 0.14%) compared to the control group (n=5). Scale bar = 500 μm in **(D)** and scale bar = 50 μm in **(E)**. S, Skin; W, Wound. Results were expressed as mean ± standard deviation (SD), and statistical significance was shown as *P < 0.05.

Moreover, to further observe whether LA can promote tissue repair by regulating macrophages activation *in vivo*, we established a skin wound healing model in mice. With the development of wound healing, daily LA injection was given to the wound tissue in Litcubanine A group, and daily PBS injection was given to the control group. By the third day after the surgery, we sacrificed the animals and found these two groups of mice showed a significant difference in the unhealed wound area (**Figure 10A**), which indicated that LA injection promoted wound healing *in vivo*. To confirm that LA inhibited inflammatory macrophages activation at the early stage of healing, we used immunohistochemistry staining to detect the local iNOS^+^ macrophages ratio. The results showed that compared to the control group (49.18% ± 10.84%), the iNOS^+^ cells ratio was lower in the LA group (12.22% ± 0.14%) (**Figures 10B–E**). These data further confirmed that LA inhibits inflammation and promotes tissue repair by inhibiting the activation of inflammatory macrophages at the early stage of injury.

## Discussion

As a professional phagocyte, macrophages are highly specialized in removing dead cells and cellular debris, and are involved in inflammation processes and inflammatory diseases (1). In recent years, the function of local macrophages in inflammatory tissues are considered to be key regulatory targets, and the related *in vivo* regulatory methods or drugs have been widely studied (1). Natural or synthetic small molecule compounds have advantages that other large molecule biological agents or drugs do not have, including their wide range of sources, controllable structure, stability, clear molecular mechanisms, and allosteric possibility.

Several studies have reported that natural products derived from medicinal plants exert significant anti-inflammatory activities and suppress the NF-κB signalling pathway, which are potential resources of anti-inflammatory candidates (30–38). In this study, we discovered and characterized a novel isoquinoline alkaloid LA that possesses an unusual C-1 methyl and a N→O group, from a medicinal plant *L. cubeba* that is widely used for the treatment of inflammatory diseases (9–11). We found LA treatment could inhibit the migration and activation of inflammatory macrophages at the injury site in a caudal fin resection model in zebrafish larvae, and in skin wound healing model in mice. Moreover, LA showed significant anti-inflammatory effects *in vitro* by inhibiting the LPS-induced activation of inflammatory macrophages *via* the NF-κB pathway, thus inhibiting the secretion of inflammatory factors including iNOS, TNF-α, and IL-1β. A molecular docking study indicated that LA targets the iNOS protein by interacting with the TRP 366 and GLU371 residues of iNOS *via* hydrogen bonds derived from the N→O group, thus further inhibiting the expression of NO in macrophages, meanwhile showing no effects on cells proliferation and apoptosis. Therefore, we have come to the conclusion that inflammatory macrophages activation pathway is an effective target for inflammation treatment, and LA is a new small molecule compound with both good biocompatibility and anti-inflammatory activity *in vivo* by inhibiting the inflammatory macrophages activation and NO expression, which may be a new pharmacophore that could prove useful for the development of novel and effective anti-inflammatory agents.

## Author’s Note

All the biosafety measurements have been adopted and the institutional safety procedures were adhered. The laboratory of our institution has biosafety level 1 (BSL-1) standard where all standards and protocols are adopted as per the guidelines of CLSI.

## Data Availability Statement

The datasets presented in this study can be found in online repositories. The names of the repository/repositories and accession number(s) can be found in the article/**Supplementary Material**.

## Ethics Statement

The animal study was reviewed and approved by Capital Medical University#2012-x-53). Association for Assessment and Accreditation of Laboratory Animal Care international (AAALAC) certification. The license number of the experimental animal is SYXK (Zhejiang) 2012-0171, and the ethic statement number was IACUC 001485.

## Author Contribution

HX isolated the compound and elucidated its structure, and contributed to the manuscript preparation in this study. YitL designed, conducted, and supervised the anti-inflammation evaluation of the compound in this study, and contributed to the manuscript preparation. GX conducted the HRMS analyses and assisted in the NMR structural determination of the compound. SL designed the compound isolation, *in vitro* study and wrote the manuscript. YL designed the *in vitro* study and wrote the manuscript. LG designed the *in vivo* study and wrote the manuscript. All authors contributed to the article and approved the submitted version.

## Funding

This work was supported by grants from the National Nature Science Foundation of China (NNSFC 81903489 to HX, 81522050 to SL) (81991504 and 81974149 to YL) (81600891 to LG), the Special Research Fund for Central Universities, Peking Union Medical College (No. 3332019080 to HX), Beijing Municipal Science & Technology Commission (Z161100000516203 to LG), Beijing Hospitals Authority Youth Programme (QML20181501), the Beijing Municipal Administration of Hospitals Clinical Medicine Development of Special Funding Support (ZYLX202121 to YL), Beijing Peak Scheme Funding Support (DFL20181501 to YL).

## Conflict of Interest

The authors declare that the research was conducted in the absence of any commercial or financial relationships that could be construed as a potential conflict of interest.

## Abbreviations

LA, Litcubanine A; *L. cubeba, Litsea cubeba*; LPS, lipopolysaccharide; Dex, dexamethasone; DMSO, dimethylsulfoxide; CC, column chromatography; IL-1β, interleukin 1 beta; TNF-α, tumor necrosis factor alpha; iNOS, inducible nitric oxide synthase; NO, nitric oxide; NF-κB, nuclear factor kappa B; IκB, inhibitor of NF-κB; IκK, IκB kinase; PGE2, prostaglandin E2; PFA, paraformaldehyde; MTD, maximum tolerated dose; dpf, days post-fertilization; hpA, hours post-amputation.

## References

[1] Liu Y Fang S Li X Feng J Du J Guo L. Aspirin Inhibits LPS-Induced Macrophage Activation *Via* the NF-κb Pathway. Sci Rep (2017) 7:11549. 10.1038/s41598-017-10720-4 28912509 PMC5599518

[2] Gordon S Taylor PR. Monocyte and Macrophage Heterogeneity. Nat Rev Immunol (2005) 5:953–64. 10.1038/nri1733 16322748

[3] Gordon S. Macrophage Heterogeneity and Tissue Lipids. J Clin Invest (2007) 117:89–93. 10.1172/JCI30992 17200712 PMC1716225

[4] Lee S Huen S Nishio H Nishio S Lee HK Choi BS. Distinct Macrophage Phenotypes Contribute to Kidney Injury and Repair. J Am Soc Nephrol (2011) 22:317–26. 10.1681/ASN.2009060615 PMC3029904 21289217

[5] Lu J Cao Q Zheng D Sun Y Wang C Yu X. Discrete Functions of M2a and M2c Macrophage Subsets Determine Their Relative Efficacy in Treating Chronic Kidney Disease. Kidney Int (2013) 84:745–55. 10.1038/ki.2013.135 23636175

[6] Murray PJ Allen JE Biswas SK Fisher EA Gilroy DW Goerdt S. Macrophage Activation and Polarication: Nomenclature and Experimental Guidelines. Immunity. (2014) 41:14. 10.1016/j.immuni.2014.06.008 25035950 PMC4123412

[7] Gasparrini M Forbes-Hernandez TY Giampieri F Afrin S Alvarez-Suarez JM Mazzoni L. Anti-Inflammatory Effect of Strawberry Extract Against LPS-Induced Stress in RAW 264.7 Macrophages. Food Chem Toxicol (2017) 102:1–10. 10.1016/j.fct.2017.01.018 28130090

[8] Lee HA Koh EK Sung JE Kim JE Song SH Kim DS. Ethyl Acetate Extract From Asparagus Cochinchinensis Exerts Anti-Inflammatory Effects in LPS-Stimulated RAW264.7 Macrophage Cells by Regulating COX-2/Inos, Inflammatory Cytokine Expression, MAP Kinase Pathways, the Cell Cycle and Anti-Oxidant Activity. Mol Med Rep (2017) 15:1613–23. 10.3892/mmr.2017.6166 PMC5364973 28260011

[9] Chinese Pharmacopoeia. Beijing: Chinese Medicine Science and Technology Publishing House (2015). p. 235.

[10] Editorial Committee of Chinese Materia Medica, State Administration Bureau of Traditional Chinese Medicine. (1999) 7:73.

[11] Zhang SY Guo Q Gao XL Guo ZQ Zhao YF Chai XY. A Phytochemical and Pharmacological Advance on Medicinal Plant *Litsea cubeba* (Lauraceae). Chin J Chin Mater Med (2014) 39:769–76. 10.4268/cjcmm20140504 25204163

[12] Lin B Zhang H Zhao X Rahman K Wang Y Ma X. Inhibitory Effects of the Root Extract of *Litsea cubeba* (Lour.) Pers. on Adjuvant Arthritis in Rats. J Ethnopharmacol (2013) 147:327–34. 10.1016/j.jep.2013.03.011 23538163

[13] Choi EM Hwang JK. Effects of Methanolic Extract and Fractions From *Litsea cubeba* Bark on the Production of Inflammatory Mediators in RAW264.7 Cells. Fitoterapia. (2004) 75:141–8. 10.1016/j.fitote.2003.11.003 15030918

[14] Zhang SY Guo Q Cao Y Zhang Y Gao XL Tu PF. Alkaloids From Roots and Stems of *Litsea cubeba* . Chin J Chin Mater Med (2014) 39:3964–8. 10.4268 /cjcmm20142014 25751947

[15] Wang LY Chen MH Wu J Sun H Liu W Qu YH. Bioactive Glycosides From the Twigs of *Litsea cubeba* . J Nat Prod (2017) 80:1808–18. 10.1021/acs.jnatprod.6b01189 28541690

[16] Wang LY Qu YH YC Li YZ Wu Li R QL G. Water Soluble Constituents From the Twigs of *Litsea cubeba* . Chin J Chin Mater Med (2017) 42:2704–13. 10.19540/j.cnki.cjcmm.2017.0119 29098825

[17] Li XT Xia H Wang LY Xia GY Qu YH Shang XY. Lignans From the Twigs of *Litsea cubeba* and Their Bioactivities. Molecules. (2019) 24:306. 10.3390/molecules24020306 PMC6359749 30654451

[18] Xia H Wang LY Xia GY Wei XH Wang YN Lin S. Chemical Constituents From Ethyl Acetate Soluble Extraction of *Litsea cubeba* . Chin J Chin Mater Med (2020) 45:5877–83. 10.19540/j.cnki.cjcmm.20200820.202M 33496127

[19] Ye Y Wang Y Yang Y Tao L. Aloperine Suppresses LPS-Induced Macrophage Activation Through Inhibiting the TLR4/NF-κb Pathway. Inflammation Res (2020) 69:375–83. 10.1007/s00011-019-01313-0 32144444

[20] Xu J Zhao Y Aisa H. Anti-Inflammatory Effect of Pomegranate Flower in Lipopolysaccharide (LPS)-Stimulated RAW264.7 Macrophages. Pharm Biol (2017) 55:2095–101. 10.1080/13880209.2017.1357737 PMC6130474 28832232

[21] Zhao Y Yang X Wu B Shang J Liu Y Dai Z. Anti-Inflammatory Effect of Pomelo Peel and its Bioactive Coumarins. J Agric Food Chem (2019) 67:8810–8. 10.1021/acs.jafc.9b02511 31318199

[22] Pase L Nowell CJ Lieschke GJ. *In Vivo* Real-Time Visualization of Leukocytes and Intracellular Hydrogen Peroxide Levels During a Zebrafish Acute Inflammation Assay. Meth Enzymol (2012) 506:135–56. 10.1016/B978-0-12-391856-7.00032-9 22341223

[23] Roehl HH. Linking Wound Response and Inflammation to Regeneration in the Zebrafish Larval Fin. Int J Dev Biol (2018) 62:473–7. 10.1387/ijdb.170331hr 29938759

[24] Elsa DG Andres SA Robin JR Matt DK Brian RC Gunilla A. Anchored Plasticity Opens Doors for Selective Inhibitor Design in Nitric Oxide Synthase. Nat Chem Bio (2008) 4:700–7. 10.1038/nchembio.115 PMC2868503 18849972

[25] Palwinder S Sukhmeet K Anuradha S Gurcharan K Rajbir B. TNF-a and IL-6 Inhibitors: Conjugates of N-Substituted Indole and Aminophenylmorpholin-3-One as Anti-Inflammatory Agents. Eur J Med Chem (2019) 174:33–4. 10.1016/j.ejmech.2017.09.003

[26] Michalczyk ER Chen L Maia MB Dipietro LA. A Role for Low-Density Lipoprotein Receptor-Related Protein 6 in Blood Vessel Regression in Wound Healing. Adv Wound Care (2020) 9:1–8. 10.1089/wound.2019.1019 PMC6918844 31871825

[27] Cheng CL Chang HM Ellis BC Huang HCY Rosalie PG. Aporphine Alkaloids and Lignans Formed in Response to Injury of Sapwood in Liriodendron Tulipifera. Phytochemistry. (1976) 15:1161–7. 10.1016/0031-9422(76)85122-9

[28] Hutchinson TH Bogi C Winter MJ Owens JW. Benefits of the Maximum Tolerated Dose (MTD) and Maximum Tolerated Concentration (MTC) Concept in Aquatic Toxicology. Aquat Toxicol (2009) 91:197–202. 10.1016/j.aquatox.2008.11.009 19124163

[29] Chi MN Builhe BL Travnickova J Luz-Crawford P Tejedor G Phan QT. Identification of Polarized Macrophage Subsets in Zebrafish. eLIFE. (2015) 4:e07288. 10.7554/eLife.07288 26154973 PMC4521581

[30] Xing HH An LJ Song ZT Li SS Wang HM Wang CY. Anti-Inflammatory Ent-Kaurane Diterpenoids From Isodon Serra. J Nat Prod (2020) 83:2844–53. 10.1021/acs.jnatprod.9b01281 32993289

[31] Kim MJ Kim DC Kwon J Ryu SM Kwon H Guo Y. Anti-Inflammatory Metabolites From Chaetomium Nigricolor. J Nat Prod (2020) 83:881–7. 10.1021/acs.jnatprod.9b00560 32163284

[32] Li YZ Chen JH Tsai CF Yeh WL. Anti-Inflammatory Property of Imperatorin on Alveolar Macrophages and Inflammatory Lung Injury. J Nat Prod (2019) 82:1002–8. 10.1021/acs.jnatprod.9b00145 30892032

[33] Valeria IR Vinicio GS Manuel RG Myrna DC Rachel M. Pharmacological Analysis of the Anti-Inflammatory and Antiallodynic Effects of Zinagrandinolide E From Zinnia Grandiflora in Mice. J Nat Prod (2021) 84:713–23. 10.1021/acs.jnatprod.0c00793 32870011

[34] Reyna ZD Leticia OC Alberto D George G Nuvia KM Rossanna RC. 22-Oxocholestane Oximes as Potential Anti-Inflammatory Drug Candidates. Eur J Med Chem (2019) 168:78–86. 10.1016/j.ejmech.2019.02.035 30798054

[35] Ji LM Qu LL Wang C Peng W Li S Yang HL. Identification and Optimization of Piperlongumine Analogues as Potential Antioxidant and Anti-Inflammatory Agents *Via* Activation of Nrf2. Eur J Med Chem (2020) 210:112965. 10.1016/j.ejmech.2020.112965 33148493

[36] Zang YD Lai FF Fu JM Li CJ Ma J Chen CJ. Novel Nitric Oxide-Releasing Derivatives of Triptolide as Antitumor and Anti-Inflammatory Agents: Design, Synthesis, Biological Evaluation, and Nitric Oxide Release Studies. Eur J Med Chem (2020) 190:112079. 10.1016/j.ejmech.2020.112079 32028140

[37] Tran QTN Wong WSF Chai CLL. The Identification of Naturally Occurring Labdane Diterpenoid Calcaratarin D as a Potential Anti-Inflammatory Agent. Eur J Med Chem (2019) 174:33–44. 10.1016/j.ejmech.2019.04.023 31022551

[38] Palwinder S Sukhmeet K Anuradha S Gurcharan K Rajbir B. TNF-a and IL-6 Inhibitors: Conjugates of N-Substituted Indole and Aminophenylmorpholin-3-One as Anti-Inflammatory Agents. Eur J Med Chem (2017) 140:92–103. 10.1016/j.ejmech.2017.09.003 28923390

