# Supplementary material for: Novel Isoquinoline Alkaloid Litcubanine A - A Potential Anti-Inflammatory Candidate
Source: Front Immunol. 2021 Jun 7;12:685556. doi: 10.3389/fimmu.2021.685556 (PMC8215673; doi:10.3389/fimmu.2021.685556)
Supplement: Supplementary file 1 [file DataSheet_1.doc]

**Novel isoquinoline alkaloid Litcubanine A- a potential anti-inflammatory candidate**

Huan Xia ^a, #^, Yitong Liu ^b, #^, Guiyang Xia ^a^, Yi Liu ^b,*^, Sheng Lin ^a,*^, Lijia Guo ^c, *^

^a^ *Key Laboratory of Chinese Internal Medicine of Ministry of Education and Beijing, Dongzhimen Hospital Affiliated to Beijing University of Chinese Medicine, Beijing 100700, P. R. China*

^b^ *Laboratory of Tissue Regeneration and Immunology and Department of Periodontics, Beijing Key Laboratory of Tooth Regeneration and Function Reconstruction, School of Stomatology, Capital Medical University, Beijing, P. R. China*

^c^ *Department of Orthodontics School of Stomatology, Capital Medical University, Beijing, P. R. China.*

**Supporting Information**

Corresponding Author

^*^ Corresponding author. Tel.: +86 10 60212110; Fax: +86 10 63017757.

E-mail addresses: [lsznn@126.com](mailto:lsznn@126.com) (S Lin) & [lililiuyi@163.com](mailto:lililiuyi@163.com) (L Liu) & [Orthoest@163.com](mailto:Orthoest@163.com) (L Guo)

## Extraction and isolation.

The air-dried twigs of *L. cubeba* (12 kg) were ground and extracted using 30.0 L 95% EtOH at ambient temperature for 3 × 48 h. The EtOH extract was concentrated *in vacuo*, and the residue was resuspended in H_2_O, then partitioned with EtOAc, to afford EtOAc- (300 g) and H_2_O (380 g)-soluble extracts. The H_2_O-soluble extract was separated using a HP-20 macroporous adsorbent resin column, then eluted successively with 10%, 30%, 70% and 95% EtOH (3000 mL each), to yield four fractions, A (165 g), B (96 g), C (63 g) and D (28 g). Fraction B was further fractionated by MPLC over a reversed-phase (RP) C18 silica gel using gradient elution (100% H_2_O to 50% MeOH) to give seven fractions (F1−F7) based on TLC analysis. Fraction F1 (2.0 g) was subjected to Sephadex LH-20 CC and eluted with MeOH to afford five subfractions (F11−F15). F12 (0.2 g) was purified by RP C_18_ HPLC (C_18_ preparative column, 5 μm, 250 × 19 mm, 254 nm, H_2_O−HOAc, 95:5) to give LA (8.6 mg).


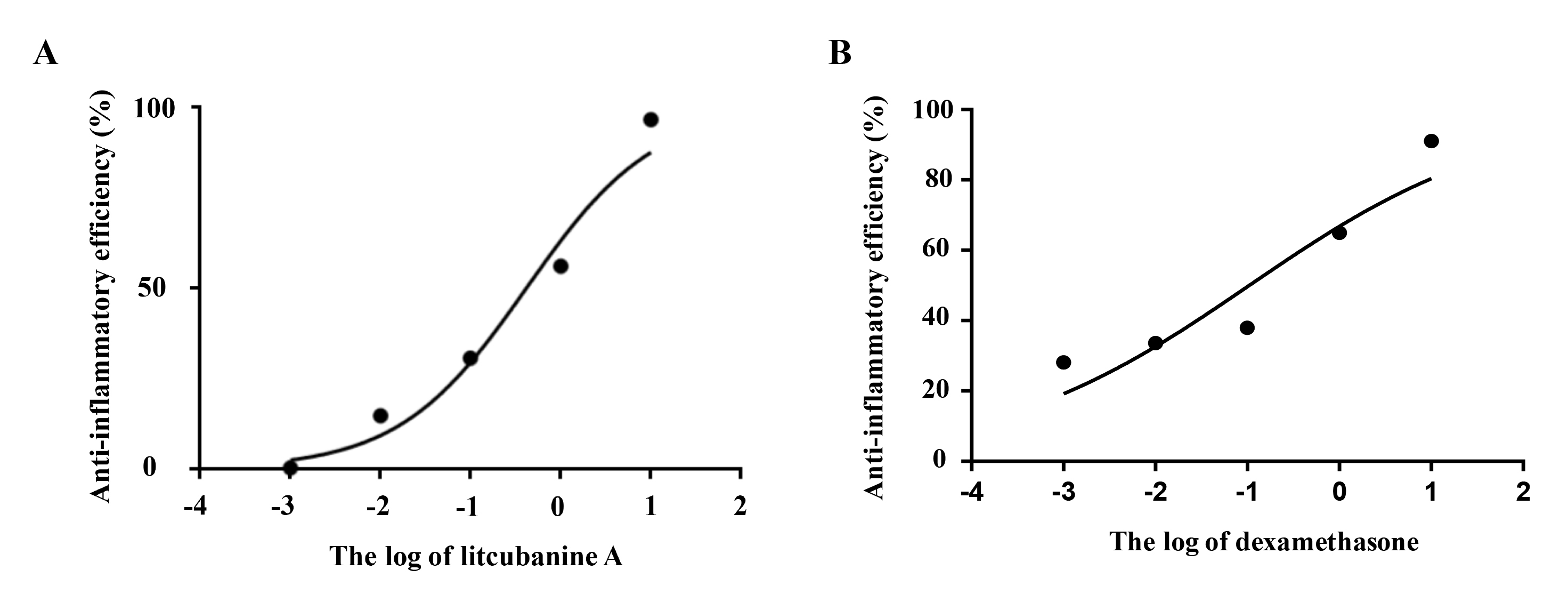


**Figure S1. The effect curves of Litcubanine A and dexamethasone** **on inhibiting LPS-induced NO production *in vitro.***

*
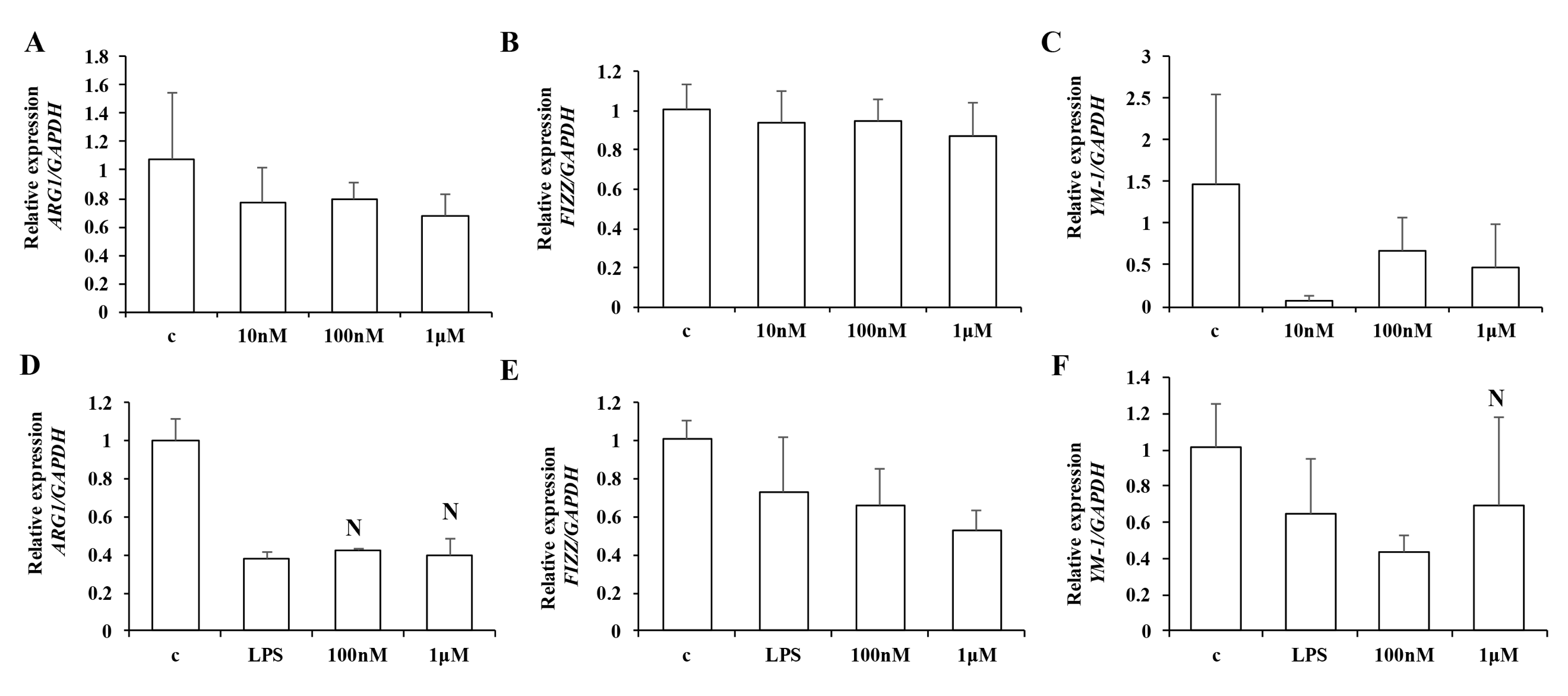
*

**Figure S2. LA treatment showed no effect on M2 macrophages activation *in vitro.***

(A-C) We treated the RAW264.7 cells with different doses of LA (10nM, 100nM, 1μM) for 24 h and performed PCR to investigate the expression levels of *ARG1*, *FIZZ* and *YM-1*, which are the classic markers of M2 macrophages. The results showed that all doses of LA showed no effect on M2 macrophages markers expression (n=3). (E-F) We further repeated the LPS-inducement experiment in this study and found that LA still had no effect on the expression of M2 macrophages markers under LPS-induced conditions (n=3). All results are representative of at least three independent experiments. Results were expressed as mean ± standard deviation (SD), and statistical significance was shown as N P>0.05.

### Figure S3. ^1^H NMR (600 MHz) spectrum of LA in DMSO-*d*_6_.


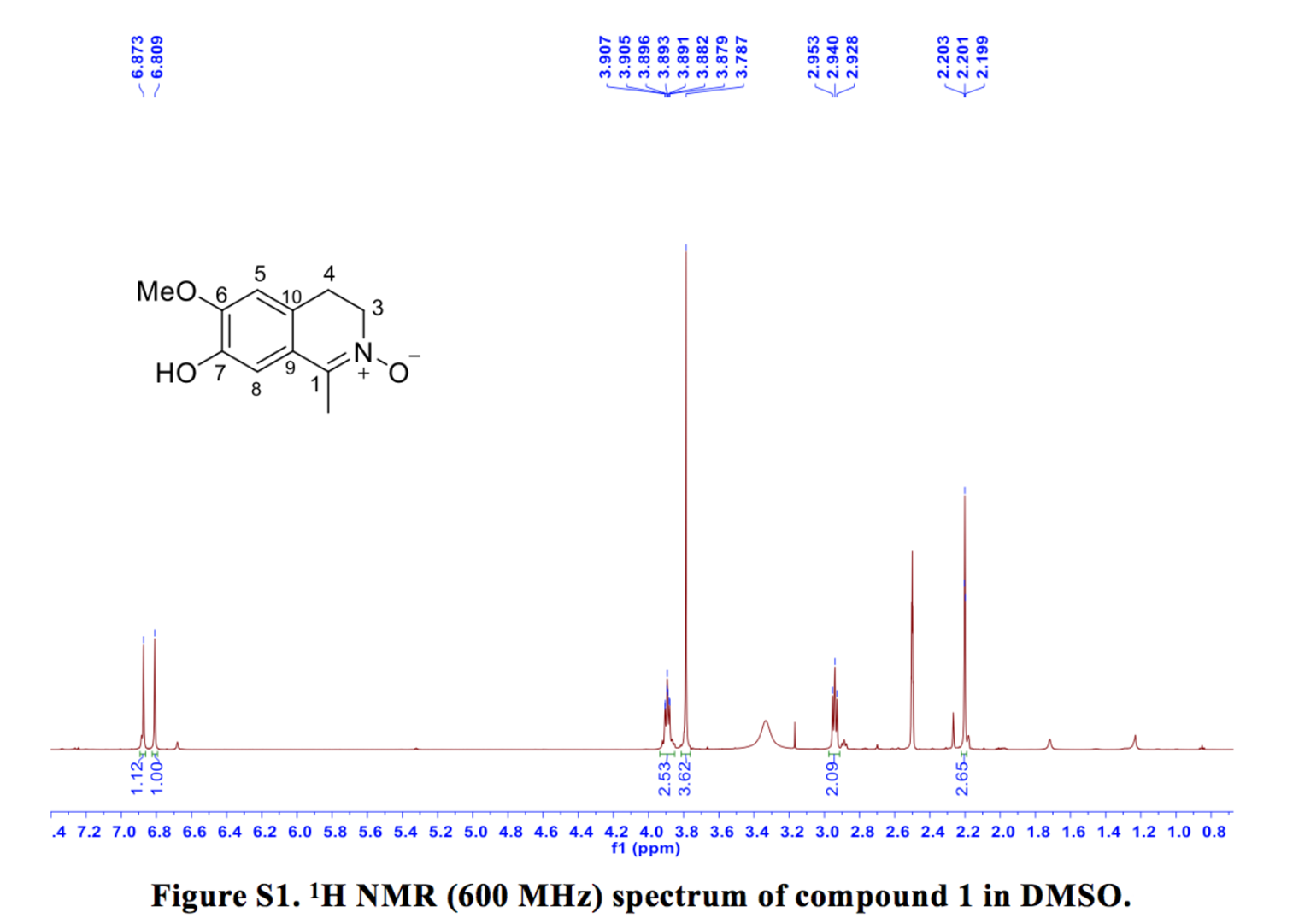


### Figure S4. ^13^C NMR (150 MHz) spectrum of LA in DMSO-*d*_6_.


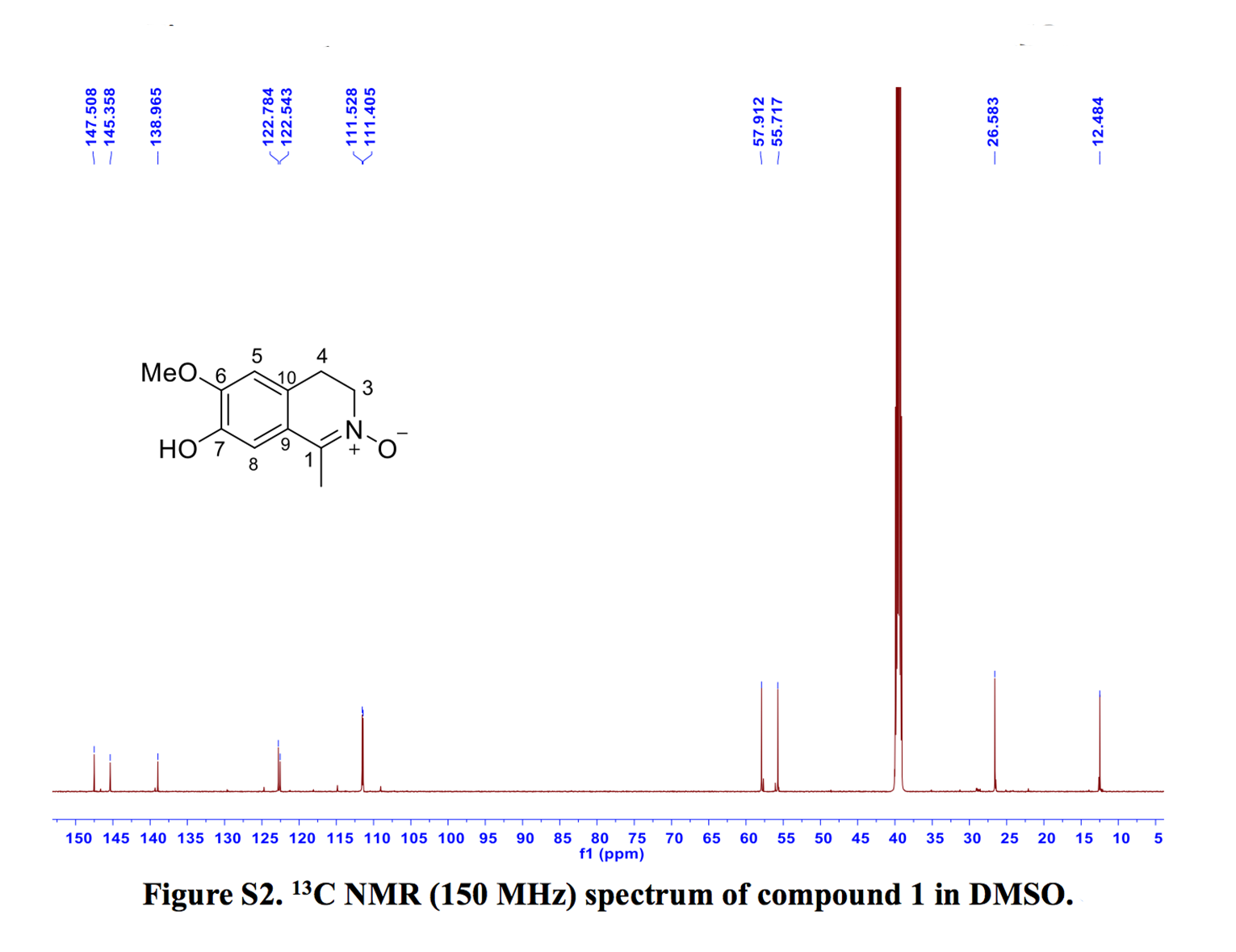


### Figure S5. HSQC spectrum of LA in DMSO-*d*_6_.


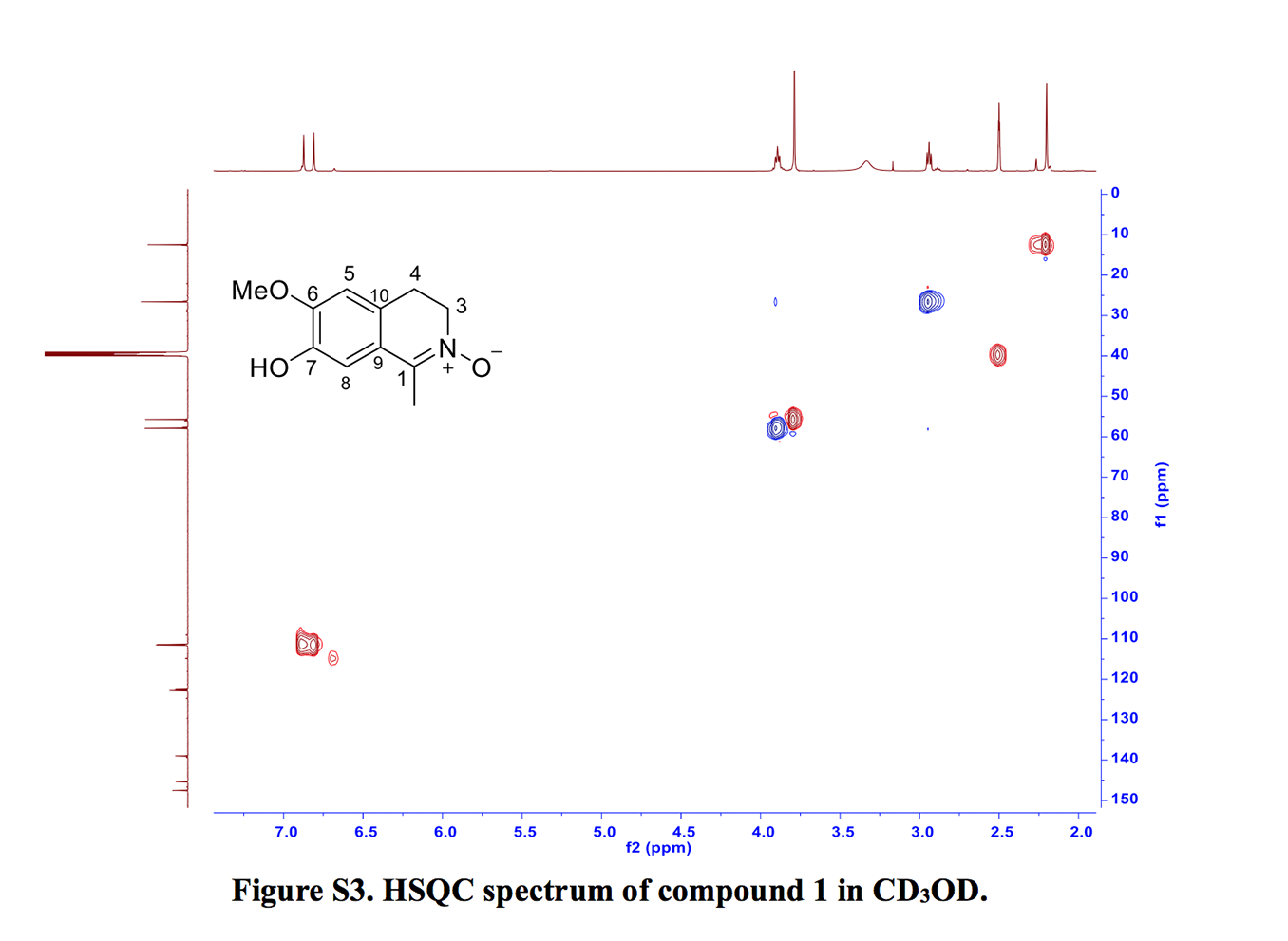


### Figure S6. ^1^H-^1^H COSY spectrum of LA in DMSO-*d*_6_.


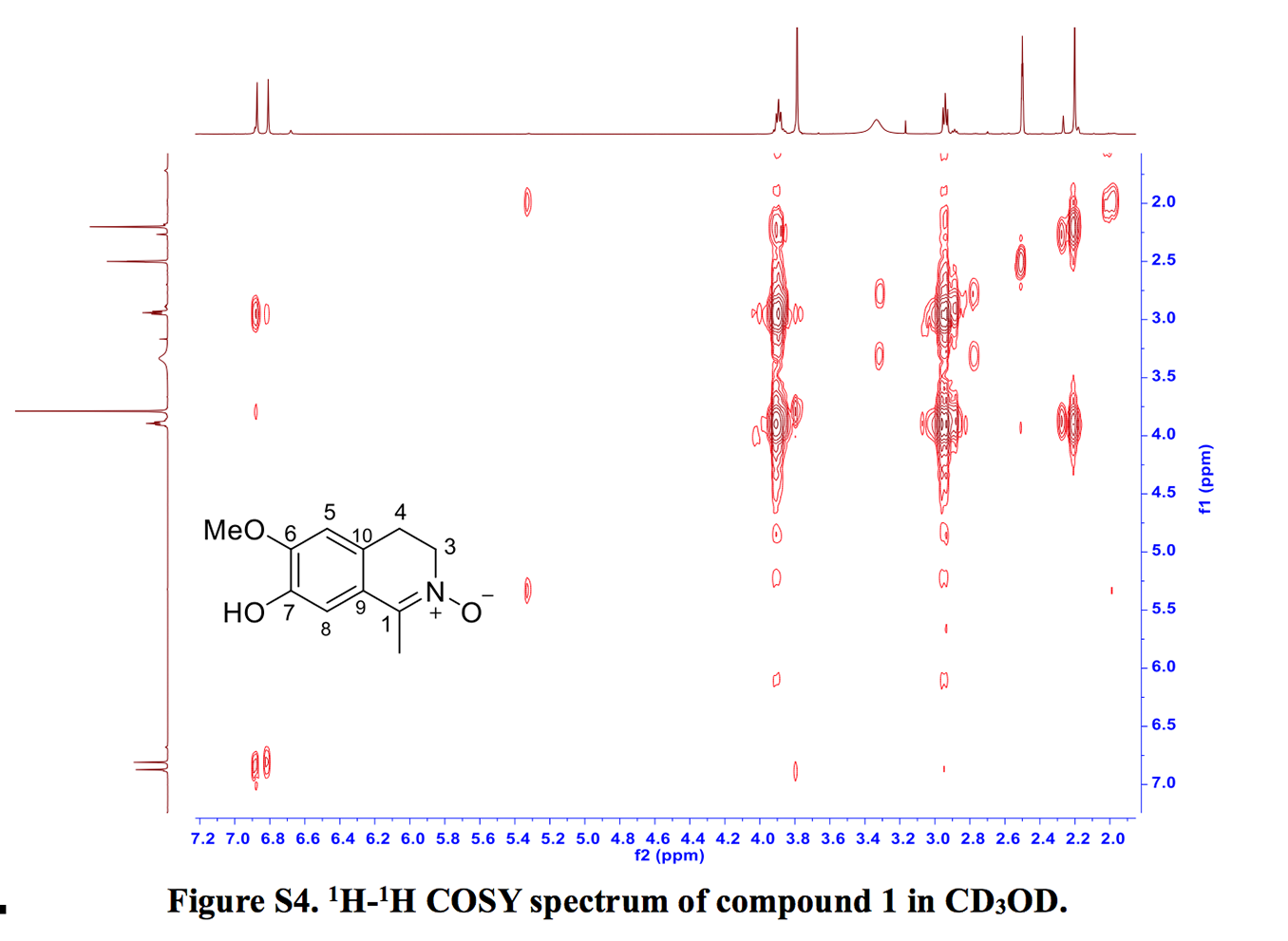


### Figure S7. HMBC spectrum of LA in DMSO-*d*_6_.


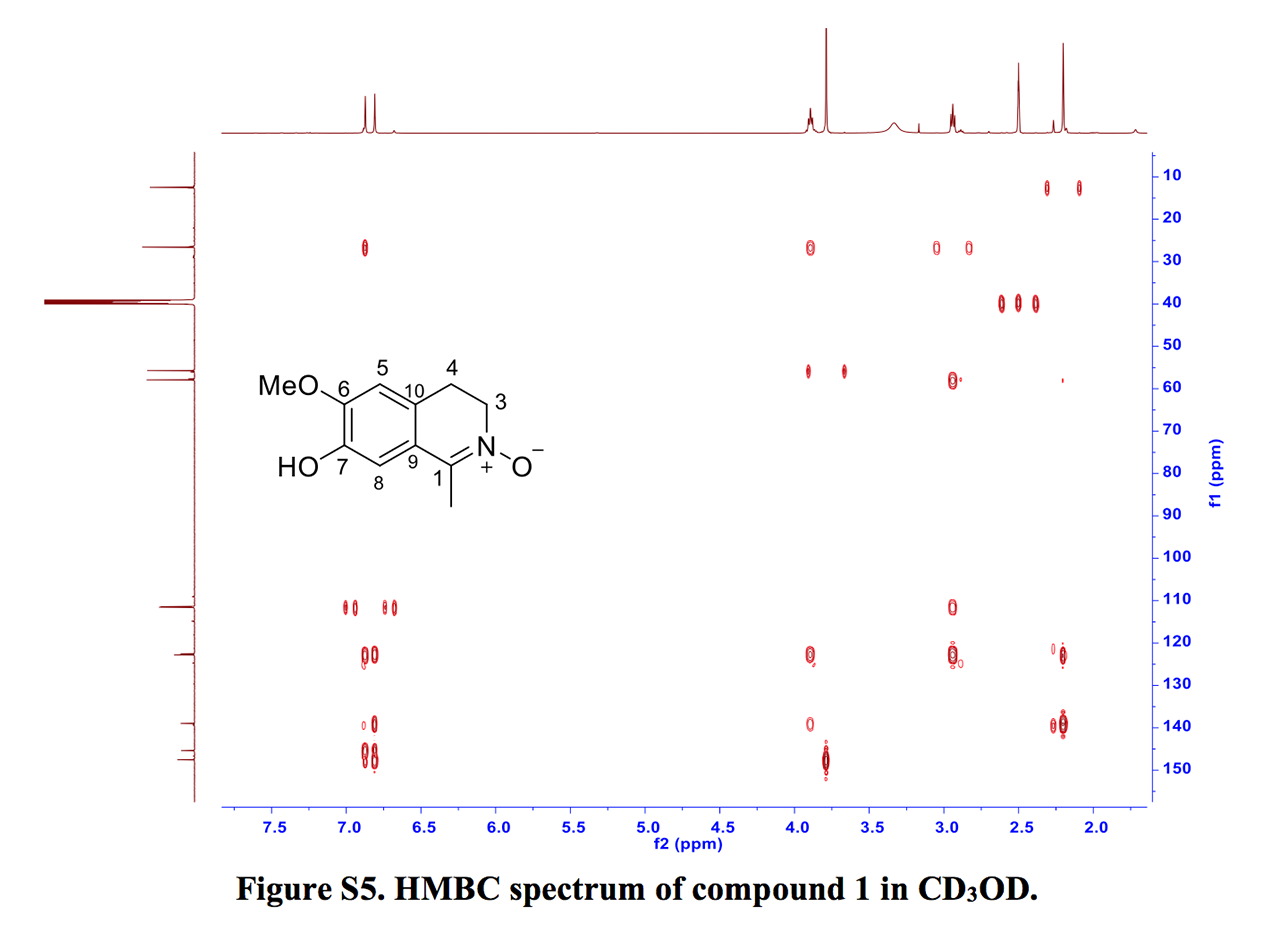


### Figure S8. All the original western blot images presented in this study.


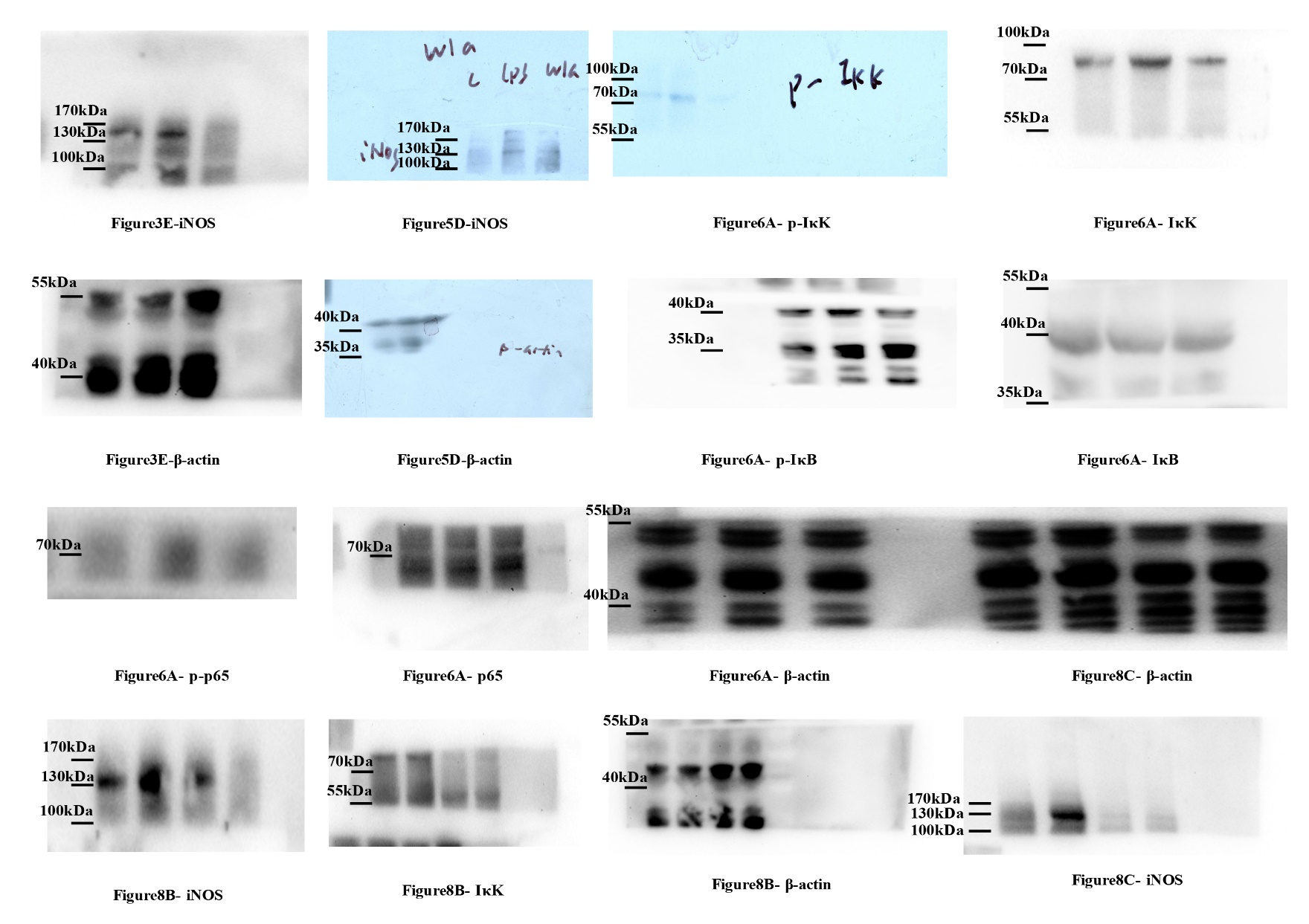


### Table. S1. Primers Used for Quantitative Real-time RT-PCR analysis.

|  | **Target** | **Forward primer** | **Reverse primer** |
| --- | --- | --- | --- |
| **All the primers used *in vitro* study.** | *iNOS* | 5’-acccaaggtctacgttcagg-3’ | 5’-cgcacatctccgcaaatgta-3’ |
|  | *TNF-α* | 5’-ccaccacgctcttctgtcta-3’ | 5’-tggtttgtgagtgtgagggt-3’ |
|  | *IL-1β* | 5’-cgctcagggtcacaagaaac-3’ | 5’-ggcaaggaggaaaacacagg-3’ |
|  | *GAPDH* | 5’-gagaggccctatcccaactc-3’ | 5’-tcaagagagtagggagggct-3’ |
| **All the primers used *in vivo* study.** | *TNF-α* | 5’-atcttcaaagtcgggtgtatg-3’ | 5’-tgtgcccagtctftctcc-3’ |
|  | *IL-1β* | 5’-gtcacactgagagccggaag-3’ | 5’-gcaggccaggtacaggttac-3’ |
|  | *iNOS* | 5’-cgccgcctgtcaatcaaata-3’ | 5’-gatggtgcacggtggaatac-3’ |
|  | *β-actin* | 5’-tcgagcaggagatgggaacc-3’ | 5’-ctcgtggataccgcaagattc-3’ |
