# Supplementary figures and images for: Novel Isoquinoline Alkaloid Litcubanine A - A Potential Anti-Inflammatory Candidate
Source: Front Immunol. 2021 Jun 7;12:685556. doi: 10.3389/fimmu.2021.685556 (PMC8215673; doi:10.3389/fimmu.2021.685556)

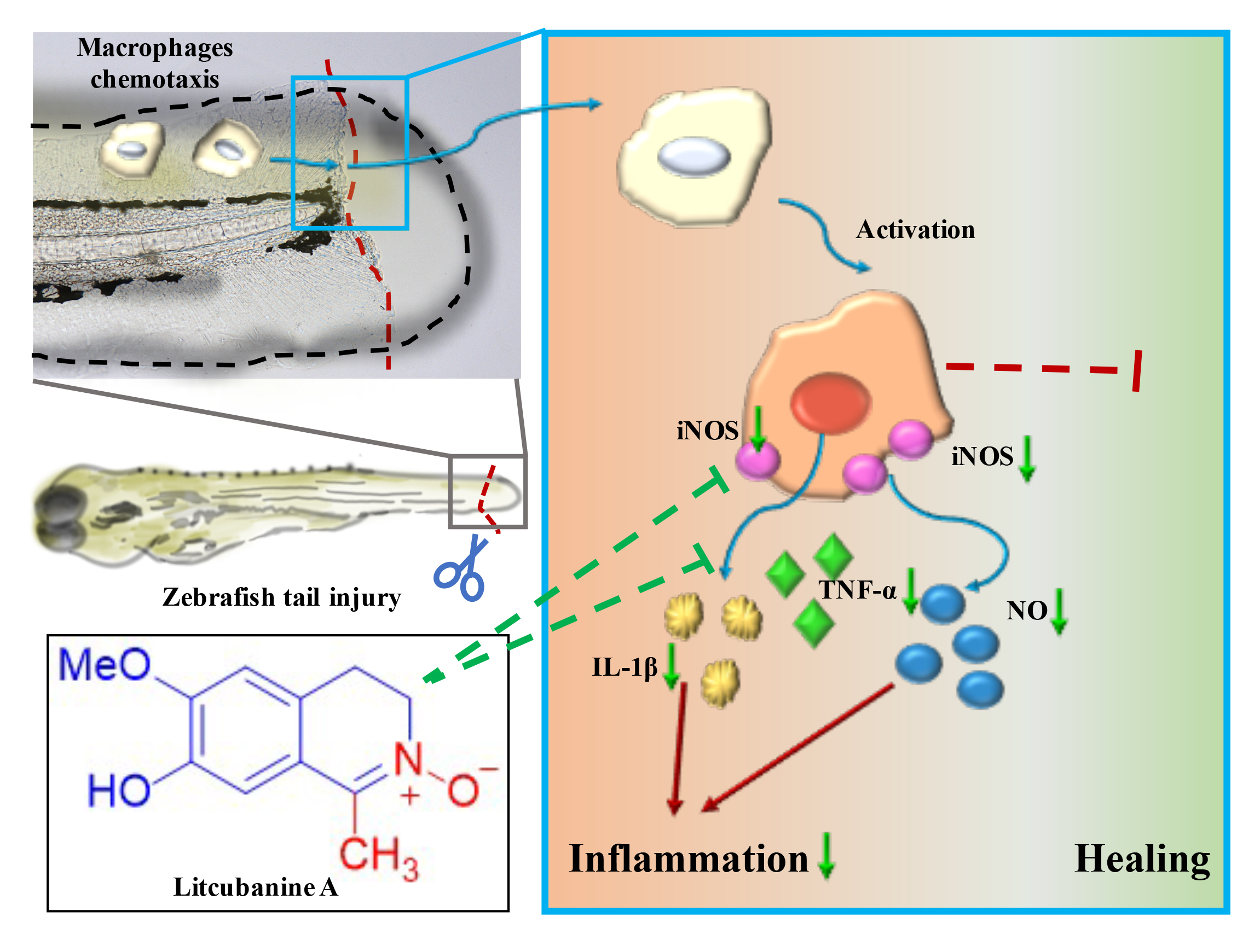

Supplement: Supplementary file 2 [file Image_1.jpeg]
